# Sleep timing in flies from “adolescence” to adulthood

**DOI:** 10.1080/19336934.2024.2448022

**Published:** 2024-12-30

**Authors:** Esther D. Domenie, Paola Cusumano, Sara Montagnese, Rodolfo Costa

**Affiliations:** a Department of Medicine, University of Padova, Padova, Italy; b Department of Biology, University of Padova, Padova, Italy; c Chronobiology Section, Faculty of Health and Medical Sciences, University of Surrey, Guildford, UK; d Institute of Neuroscience, National Research Council (CNR), Padova, Italy; e Department of Biomedical Sciences, University of Padova, Padova, Italy

**Keywords:** *Drosophila melanogaster*, locomotor activity, sleep timing, age, light

## Abstract

The aim of the present study was to assess sleep timing in *Drosophila melanogaster* at different ages, within the setting of an enforced schedule of varying light-dark stimuli, simulating light exposure variations between four typical office *working days* and one *free day* spent outside by a human, for a total of 30 days. Locomotor activity recording started when male flies were 3 days old. Flies exhibited a bimodal activity pattern, with a morning and an evening peak, and clear anticipation of the *lights on* and *lights off* transitions. From experimental day 10 (i.e. 12-day-old flies) onwards, a decrease in activity counts/increase in sleep amount were observed. On *free days*, a rise in activity counts and a reduction in sleep amount during the *lights on* interval was observed and was also present, albeit less obvious, on the subsequent *working day* during the *lights off* interval. A progressive delay in sleep onset was observed in the first days of the experiment, peaking on day 4 (i.e. 6-day-old flies), after which sleep onset timing gradually advanced. A delay in sleep offset was also observed for the first 13 days of the experiment, after which sleep offset stabilized. In conclusion, ‘adolescent’ flies exhibited changes in sleep timing that were reminiscent of those of human adolescents.

## Introduction

*Drosophila melanogaster* is often utilized as a model to study sleep, and circadian sleep regulation in particular [1,2]. This is because there are times in which *Drosophila melanogaster* exhibits a state of quiescence characterized by increased arousal threshold and rapid reversibility in response to sufficiently strong stimuli [3,4]. Features of both circadian and homoeostatic regulation of sleep have been documented in *Drosophila melanogaster* [1], together with changes in neuronal activity which are reminiscent of those observed in human sleep [1,5]. Furthermore, several genetic and molecular sleep regulators are conserved from *Drosophila* to humans [6,7]. Finally, its relatively short life-span makes *Drosophila* a convenient model to study age-associated changes in sleep [8].

In humans, sleep timing varies with age, with a prominent delay in sleep timing during adolescence [9–12]. This has been attributed to changes in both homoeostatic and circadian sleep regulation [10,11], which can be exacerbated by poor light-dark hygiene and social factors [13,14]. Interestingly, this delay in sleep timing does not seem to be specific to humans but has also been observed in the marmoset [15] and other vertebrates, including the rhesus monkey, degu, rat and mouse [11].

The aim of the present study was to assess sleep timing in *Drosophila melanogaster* at different ages, within the setting of an enforced schedule of varying light-dark stimuli simulating human social structures. This had been originally designed as a preparatory experiment for subsequent shift work simulations.

## Material and methods

### Experimental design

**Figure 1.**
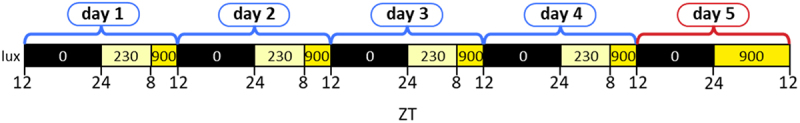
Experimental light:dark schedule over one complete module, i.e. 4 *working days* followed by a *free day*.

The experiment lasted 30 days for a total of six 5-day modules (i.e. four *working days* followed by one *free day*; Figure 1). Light intensity varied during the experiment, with a view to simulate light exposure variations between a typical office *working day* and a *free day* spent outside (overcast conditions) by a human. Thus, on *working days* light intensity was lower (230 lux) for the first 8 hours after lights on (ZT0-ZT8, 09:00–17:00 clock time); this was followed by 4 hours of brighter light (900 lux; ZT8-ZT12, 17:00–21:00 clock time) (Figure 1). Every fifth day, flies were exposed to 12 hours of bright light (900 lux; ZT0-ZT12, 09:00–21:00 clock time) (Figure 1). Both light intensities utilized are higher than those fruit flies prefer in natural settings [16,17], and can therefore be considered, at least to some extent, as stressors. The light source was a set of halogen photo-optic lamps, which were switched on in different numbers to obtain the 230 and 900 lux intensities, monitored by a luxmeter.

### Fly stocks and maintenance

Wild-type Canton-S *Drosophila melanogaster* fertilized eggs were collected within a 30 minutes interval and placed in vials (25 mm diameter by 94 mm length) with 5 ml standard cornmeal medium (1 litre: 50 g inactivated yeast powder, 8.5 g agar, 72 g cornmeal, 79.3 g sucrose, 13.5 ml Nipagin in 75% ethanol) at a controlled density of approximately 50 eggs per vial. Adult flies were collected after hatching, raised on standard cornmeal medium and maintained in a rectangular 12 h:12 h light:dark (LD 12:12, 900 lux) cycle at 23°C.

### Locomotor activity recording and analysis

Three-day-old male flies (*n* = 128) were housed in glass tubes (5 mm diameter by 65 mm length), 1 fly per tube; flies were transferred to fresh food (1 litre water, 44 g inactivated yeast powder, 12 g agar, 44 g sucrose, 10 ml Tegosept 2% ethanol) tubes every 5 days, with no anaesthesia by author PC. Locomotor activity was monitored with the *Drosophila* Activity Monitoring (DAM) single beam system (TriKinetics, Waltham MA). TriKinetics monitors were housed inside a thermostatic room where temperature (23°C) and humidity (70%) were kept constant. Locomotor activity was recorded from *Zeitgeber* Time 12 (ZT 12, 21:00 clock time of Day 1, lights off) until the morning of day 30 (11:00 clock time, ZT2). Activity was measured as the number of beam crosses (activity counts) recorded at 1-min intervals (Supplementary Table S1, raw data file).

Raw data actograms were obtained for each fly using the ActogramJ plug in for ImageJ [18], and locomotor activity was visually evaluated to check for obvious abnormalities. Rhythmicity was then assessed by Chi-square periodogram analysis [19] using the ActogramJ plug in for ImageJ [18], with a cut off *p* value of 0.05. Flies that did not show significant rhythmicity or died during the experiment were excluded from further analyses.

Activity counts were summated per day, separately for lights on and lights off. One-min activity counts were summated into 30-min bins [8]. Activity profiles were then averaged across the four *working days* of each module. *Free days* were analysed separately.

### Sleep analysis

A period of inactivity (i.e. 0 activity counts) lasting a minimum of 5 minutes was qualified as sleep [4,20]. Activity counts were summated into 5-minute bins to calculate the sleep amount in minutes. To create daily sleep profiles, the amount of sleep per 30 minutes was then computed. Sleep profiles were averaged per module across the four *working days* and *free day* profiles were kept separate.

The number and duration of sleep bouts (i.e. uninterrupted series of one or more 5-minute activity bins with 0 activity) and the timing of the longest sleep bout (i.e. the start time of the longest sleep bout) per day were also calculated separately for the *lights on* and *lights off* intervals.

For purposes of this study, sleep onset after lights off (ZT12, 21:00 clock time) was defined as the start time of the first sleep bout lasting at least 25 minutes. Sleep offset before lights on was defined as the end time of the last sleep-bout lasting at least 25 minutes before lights on (ZT0, 09:00 clock time). Both sleep onset and offset analyses were repeated also setting the sleep bout length at 15 and 35 minutes.

### Sample size estimate and statistical analysis

Published results [8] suggest that the observed difference in average duration of a sleep bout in flies aged 3 days (mean ± SD: 50 ± 27, from Figure 1 in [8] with SEM converted into SD based on *n* = 30) *versus* 30 days (75 ± 54, from Figure 1 in [8] with SEM converted into SD based on *n* = 30), with α = 0.05, power 90/95% and r (correlation between samples) 0.3/0.5 can be detected by use of 40–60 flies [estimates made by G*Power [21]]. Considering that two additional variables are present in our study (i.e. the change in light intensity during *working days* plus the change in light intensity on *free days*, for which there are no precedents in the literature), a sample size of *n* = 128 was considered adequate, also to encompass any minor losses in the final number of flies studied.

Replicate experiments were not carried out. However, a subset of analyses of the main findings (*vide infra*) were repeated and compared in two randomly generated subsamples of approximately equal size (57 and 56 flies out of the final sample of 113, *vide infra*).

Data are presented as mean ± SEM or mean ±95% Confidence Intervals (CI), unless otherwise specified. The distribution of variables was tested for normality using the Shapiro-Wilk’s *W* test. Variations in locomotor activity/sleep indices over time (days or modules) were analysed by repeated measures ANOVA (by group, where needed, with *post hoc* Scheffé test in both instances) or the nonparametric Friedman test, as appropriate. Paired t or Wilcoxon matched pairs tests were utilized to compare locomotor activity/sleep indices in *free* versus *working day* averages. Analyses were performed by Statistica, version 14.0.0.15 (TIBCO, Palo Alto, CA, USA).

## Results

Out of the initial 128 flies, 14 died during the experiment and one did not show a significant rhythmicity pattern. Thus, the final sample encompassed 113 flies. There is no reason to hypothesize that exclusions would have resulted in bias, as only one fly was removed because of lack of rhythmicity, and deaths are most likely related to manipulation (mainly transfer of flies into tubes with fresh food), and therefore they are operator- rather than fly-dependent.

### Activity and sleep patterns over time, by module

**Figure 2.**
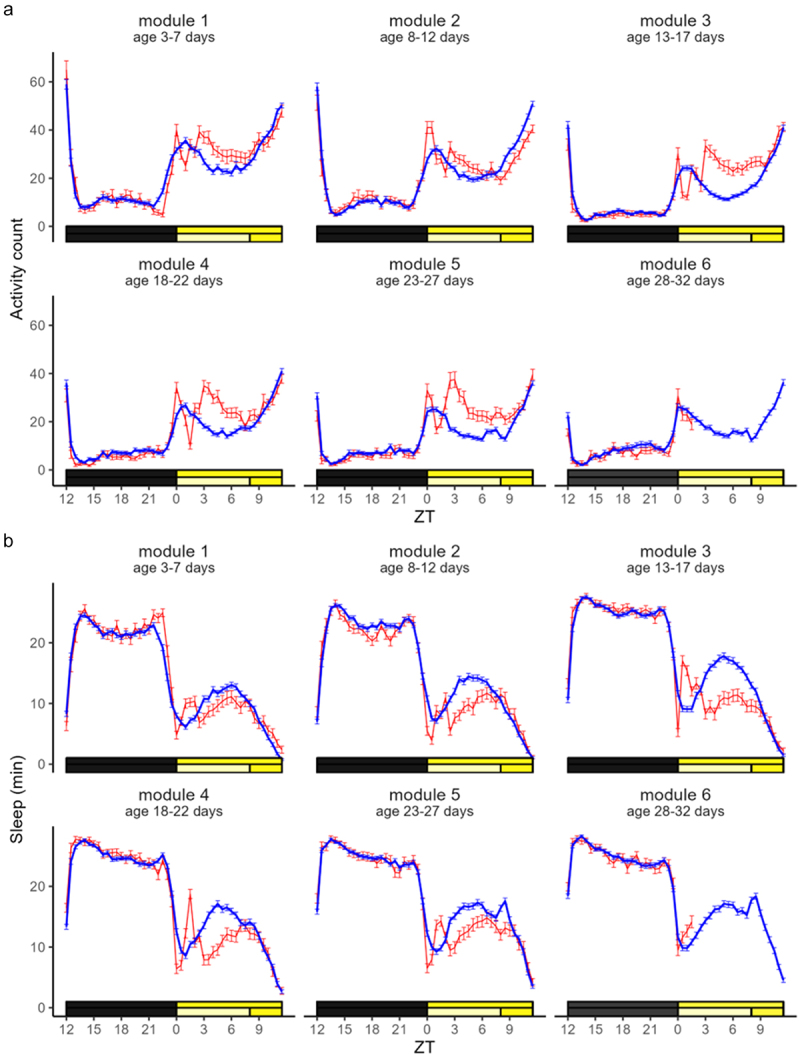
Activity and sleep profiles, by module. Activity counts (a, mean ± SEM) and minutes of sleep per 30 minutes (b, mean ± SEM) over time expressed as *zeitgeber* time (x axis) for *working days* (average within each module in blue) and *free days* (in red) in *n*=113 flies. The light:dark schedule for *working* (bottom bar) and *free days* (top bar) is indicated on top of the x axis (black: darkness; light yellow: 230 lux; bright yellow: 900 lux). *Note: for logistic reasons, recordings ended on the morning of day 30 (11:00 clock time, ZT2), thus the free day of module 6 was shorter than the others.*

Flies exhibited a bimodal activity pattern, with a morning and an evening peak and clear anticipation of the *lights on* and *lights off* transitions (Figure 2a). Sleep was observed mostly during the *lights off* interval, plus in the middle of the day, the so called *siesta* (Figure 2b). On visual analysis, activity levels were reduced, and sleep was increased in modules 3–6 when compared to modules 1–2, both during *lights on* and during *lights off* (Figure 2 and Supplementary Figure S1). This was confirmed on repeated measures ANOVA *(activity*, time: F = 107.9, p < 0.001; light/dark: F = 262.7, p < 0.001; time*light/dark: F = 6.673, p < 0.001; *sleep*, time: F = 131.6, p < 0.001; light/dark: F = 621.5, p < 0.001; time*light/dark: F = 6.425, p < 0.001). On *free days*, the morning activity peak exhibited ‘bimodal’ features, with the initial, pronounced increase in locomotor activity most likely being due to a strong light intensity-related startling effect (Figure 2). The subsequent *siesta* was delayed and characterized by a higher level of locomotor activity compared to *working days* (in red, in Figure 2a). This was also reflected in the sleep profile (in red, in Figure 2b). No obvious differences between *working* and *free days* were observed during the *lights off* interval.

### Activity and sleep patterns over time, by day

**Figure 3.**
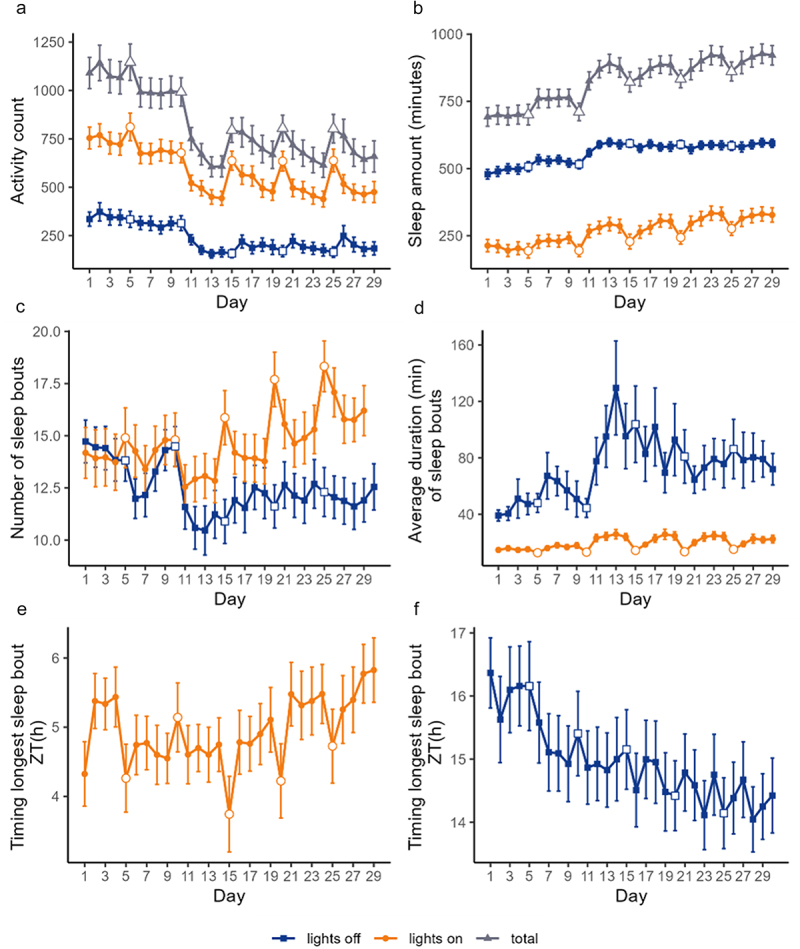
Sleep indices over time, and by lighting conditions. Activity counts (a, mean ± 95% CI) and sleep amount (b, mean ± 95% CI) overall (grey plots), during *lights on* (orange plots) and during *lights off* (blue plots). Number of sleep bouts (c, mean ± 95% CI) and average sleep bout duration (d, mean ± 95% CI) during *lights on* (orange plots) and during *lights off* (blue plots). Timing of the longest sleep bout during *lights on* (e, mean ± 95% CI) and during *lights off* (f, mean ± 95% CI). *Free days* are marked as open symbols (grey triangles overall, orange circles for *lights on* and blue squares for *lights off*) in each plot.

The daily activity count and sleep amount varied significantly over time (*overall*: activity counts: F = 47, *p* < 0.001; sleep amount: F = 57, *p* < 0.001; *lights on*: activity counts: F = 39.02, *p* < 0.001; sleep amount: F = 33.27, *p* < 0.001; *lights off*: activity counts: F = 31, *p* < 0.001; sleep amount: F = 54.32, *p* < 0.001) (Figures 3a,b). On visual analysis, from day 10 onwards (i.e. 12-day-old flies), a decrease in activity counts and a parallel increase in sleep amount were observed (Figures 3a,b). This was substantiated by the fact that *post hoc* analyses for daily activity counts and sleep amount were significant mostly for days 1–10 *versus* days 12–29 (Supplementary Tables S2A-F). The number of sleep bouts, the average sleep bout duration and the timing of the longest sleep bout (i.e. the start time of the longest sleep bout, calculated separately for the *lights on* and *lights off* intervals) also varied significantly over time (*lights on*: n bouts: F = 8.6, *p* < 0.001; average duration: F = 20.3, *p* < 0.001; timing: F = 4.9, *p* < 0.001; *lights off*: n bouts: F = 9.4, *p* < 0.001; average duration: F = 7.8, *p* < 0.001; timing: F = 5.1, *p* < 0.001) (Figures 3c-f).

On visual analysis, around day 11, an obvious change seemed to occur, with a decrease in the number of sleep bouts and a parallel increase in their duration during the *lights off* interval. The phenomenon peaked around days 12–13, which were also significantly different from most earlier days on *post hoc* analyses (Supplementary Tables S3A-B).

On *free days*, a rise in activity counts (paired t test: *p* < 0.001) and a reduction in sleep amount (*p* < 0.001) during the *lights on* interval was observed and was also present, albeit less obvious, on the subsequent *working day* during the *lights off* interval (Figures 3a,b). On *free days*, the number of sleep bouts was higher (*p* < 0.001), sleep bout duration shorter (*p* < 0.001) and timing of the longest sleep bout earlier (*p* < 0.001) during *lights on* (Figures 3c-e).

### Sleep timing over time, by day

**Figure 4.**
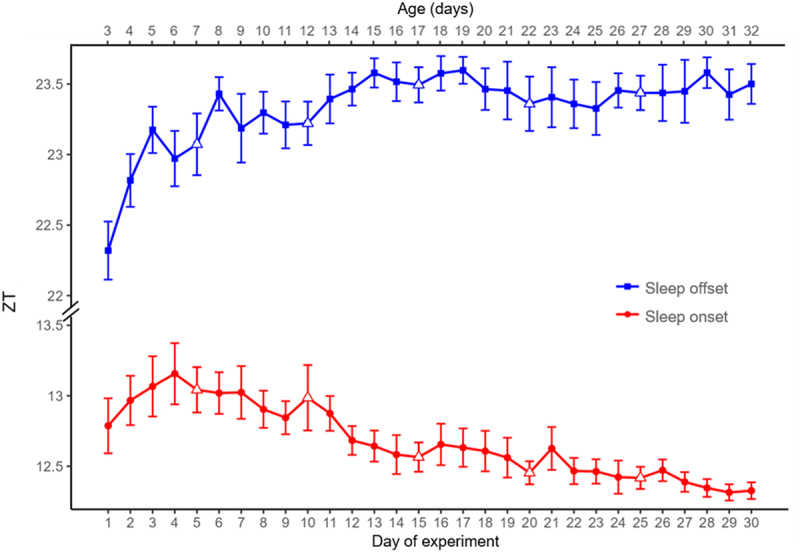
Sleep onset and offset over time, by day of experiment and fly age. Sleep onset, red circles (mean ± 95% CI) and offset, blue squares (mean ± 95% CI); red/blue empty triangles mark *free days*; time is expressed as ZT, decimal hour.

Sleep onset and offset significantly varied over time (sleep onset: F = 21, *p* < 0.001; sleep offset: F = 13, *p* < 0.001) (Figure 4). These results were unchanged when analyses were repeated setting the sleep onset/offset criteria to the first/last sleep bout duration of 15 and 35 minutes.

On visual analysis, there was a progressive delay in sleep onset in the first days of the experiment, peaking on day 4 (i.e. 6-day-old flies), after which sleep onset timing gradually advanced. The difference was 22 minutes between day 1 and day 4, and almost 1 hour between day 4 and the final days of the experiment. This was substantiated by the fact that *post hoc* analyses were significant mostly for days 2–7 *versus* days 20–30 (Supplementary Table S4A). Further, a slight delay in sleep onset was visible on days 16, 21 and 26, all after a *free day* (Figure 4).

On visual analysis, there was a delay in sleep offset for the first 13 days of the experiment (albeit with some significant oscillations and slope changes), after which sleep offset tended to stabilize. *Post hoc* analyses were significant for days 1 *versus* days 3, 5–30 and day 2 *versus* days 13, 14, 16, 17 and 28 (Supplementary Table S4B).

The above analyses on sleep onset and sleep onset evolution over time were repeated in two random subsamples of 56 and 57 flies. The results for the overall (*n* = 113) group were confirmed, with no significant differences being detected between the two subsamples (Supplementary Figure S2).

## Discussion

In humans, sleep timing delays during adolescence, with a delay in sleep onset that has been attributed, at least in part, to ongoing development of the circadian and homoeostatic systems and their interactions [10–12]. In addition, poor light-dark hygiene and social and environmental factors including social engagements, increasing academic pressure, extracurricular activities together with reduced parental monitoring of bedtimes may also contribute [10,13,14,22]. Here we observed age-related changes in sleep timing in *Drosophila melanogaster*, which is commonly used as a model organism to study circadian rhythms and related sleep phenotypes [1,23,24]. Despite obvious differences between insects and mammals, it should be highlighted how remarkable similarities have been reported between both circadian [1] and homoeostatic [5,20] sleep regulation in the fruit-fly and in humans. In addition, both mammals and invertebrates such as *Drosophila melanogaster* exhibit transitions between distinct sleep stages, which are defined by electrophysiological and behavioural indices [25]. Further, flies and mammals share a degree of endocrinology and ageing physiology [26], and some stages of fly development are reminiscent of human adolescence, with an evolving and integrated interaction between nutrient sensing, insulin signalling and juvenile hormone levels [27]. Thus, while our use of the term ‘adolescence’ is clearly a simplification, it is aimed at highlighting common sleep-wake features between human adolescents and young fruit-flies. Fruit-flies represent a manageable and very well studied model of both circadian rhythmicity and sleep, and may be utilized in future studies to uncover the physiology of changes in sleep-wake timing during the lifespan.

Our experiments were conducted within the setting of an enforced schedule of varying light-dark stimuli designed to simulate light exposure in humans in *working* versus *free days*. This should not be interpreted as a strict comparator to human weeks but more as an attempt to model social structures. Similarly, our *free* day mimics a weekend spent outside in relatively bright light conditions, which is obviously only one of several weekend options for a human adolescent.

Sleep amount increased with age. Concurrently, the number of sleep bouts decreased while average sleep bouts duration increased. Similar observations were reported by Koh et al. [8], in males (2 Canton-S strains) and in the first 25 days of age, in a 60-day study of fly sleep behaviour. A progressive increase in sleep duration with age, but no significant difference in the number and duration of sleep bouts, was also reported by Bushey et al. [28], who monitored sleep behaviour in wild-type 2 Canton-S flies and three short sleeping mutants from 10–120 days of age. At odds with our findings, a cross-sectional study by Vienne et al. [29] found a decrease in total sleep amount and an increase in sleep fragmentation with age, comparing 8-, 20- and 35-day-old Canton-S flies, rather than continuous profiles like in our and other studies. Increased sleep duration was reported in younger Canton-S and *iso31* flies compared to those studied here (from 1 to 5–7 days [25, 30]). The above discrepancies are most likely related to differences in fly strains (mostly Canton-S but cultured in different labs, and thus most likely exhibiting some degree of variability in behaviour), flies’ age, length of the observations, and experimental set-ups, with the present study being fairly unique in terms of the light regimes the flies were exposed to both on *working* and *free* days. However, our results will need to be confirmed in studies with a similar set-up and adolescent and older flies starting the experiment on the same day, in parallel. These will unmask any time-in-assay effects, which might have contributed to some of the differences observed between our and previously published findings.

Sleep timing varied significantly over time, with a progressive delay in sleep onset during the first days of the experiment, reaching its peak around 6 days post-eclosion. From then onwards, sleep onset gradually advanced. This resembles the delay in sleep onset observed in humans and several other mammals around puberty/sexual maturation [11,12,15], and the subsequent slower and gradual return to earlier sleep onsets [12]. Sleep offset timing delayed, albeit at a varying slope, until around 15 days post-eclosion, subsequently stabilizing, which is also reflected in the shorter sleep amount observed in younger flies. This may, to some extent, mimic what happens to human adolescents during the school/busy days, with *lights on* both entraining the circadian clock and almost acting as an alarm clock. Over recent years, the implications of school start times in terms of sleep duration and quality, academic performance and the health of adolescents and young adults have been widely investigated [12,22,31–34]. Of interest, changes in sleep architecture and sleep duration in the early/juvenile phase of the fruit fly development have been associated with abnormalities in brain development and behaviours such as courtship [30]. This suggest that modelling the effect of social structures on fly sleep duration may be of interest also beyond its obvious parallels with human sleep-wake phenotypes.

Interestingly, light intensity also seemed to influence the amount of sleep and sleep timing, with a decrease in the amount of sleep and a small delay in sleep timing on *free days*, during which light intensity was higher. Furthermore, on *free days* flies showed a split morning peak, affecting the start of their siesta. This phenomenon, which is likely to be light-related, became more obvious across modules, which could relate to increased light sensitivity/escape behaviour with ageing. The increase in activity and decrease in sleep is not entirely in line with previous studies reporting decreased activity levels and increased length of siesta with higher light intensities [16,17]. However, there were profound differences in the experimental set ups, length of the recordings and light intensity profiles utilized in the previously published studies compared to ours, which has a somewhat unique cyclic structure, and in which higher light intensities could have also acted as a stressor.

Our study has several limitations. The four-plus-one module to mimic a human working week is obviously arbitrary. However, given that a ‘day’ can be no different from 24 hours (in this respect humans and flies are similar) and the lifespan of a fly is 2–3 months (in laboratory conditions), the choice seems a reasonable compromise to both guarantee a preserved proportion between *working* and *free* days, and an adequate number of modules for purposes of analyses. Therefore, our experimental set-up should be interpreted as an attempt to simulate human social structures rather than strictly reproduce human weeks. The same applies to the chosen light set-up for the *free days*, which mimics the weekend of a human who goes on a trip in the open air; different and even alternate set-ups to also mimic ‘lazy’ weekends spent inside may be used in future studies. Finally, the use of males only (the main reason for which is that eggs laying can impinge on signal recording of locomotor activity) and of a single strain obviously impinges on the generalizability of our observations. Finally, virtual replicates generated by subsampling cannot replace independent biological replicates, which is a further limitation of our study.

In conclusion and despite the above limitations, our results hint at a delay in sleep onset up until 6 days post eclosion, after which sleep onset advances. As *Drosophila melanogaster* has proven to be a useful model to study sleep [35,36], the observed sleep timing pattern of ‘adolescent’ flies, which is reminiscent of that of adolescent humans, could be valuable to study the mechanisms underlying the latter.

## Supplementary Material

FliesAge_Suppl_Table2.docx

FliesAge_Suppl_Table4.docx

FliesAge_Suppl_Table3.docx

FliesAge_Suppl_Table1_raw_data.xlsx

FliesAge_Suppl_Figures1_and_2.docx
