# Supplementary material for: Sleep timing in flies from “adolescence” to adulthood
Source: Fly (Austin). 2024 Dec 30;19(1):2448022. doi: 10.1080/19336934.2024.2448022 (PMC11702927; doi:10.1080/19336934.2024.2448022)
Supplement: FliesAge_Suppl_Table2.docx [file KFLY_A_2448022_SM2380.docx]

| **Day** | **1** | **2** | **3** | **4** | **5** | **6** | **7** | **8** | **9** | **10** | **11** | **12** | **13** | **14** | **15** | **16** | **17** | **18** | **19** | **20** | **21** | **22** | **23** | **24** | **25** | **26** | **27** | **28** | **29** |
| --- | --- | --- | --- | --- | --- | --- | --- | --- | --- | --- | --- | --- | --- | --- | --- | --- | --- | --- | --- | --- | --- | --- | --- | --- | --- | --- | --- | --- | --- |
| **1** |  | 1.0000 | 1.0000 | 1.0000 | 1.0000 | 1.0000 | 0.9999 | 0.9999 | 1.0000 | 1.0000 | 0.0000 | 0.0000 | 0.0000 | 0.0000 | 0.0001 | 0.0000 | 0.0000 | 0.0000 | 0.0000 | 0.0005 | 0.0000 | 0.0000 | 0.0000 | 0.0000 | 0.0004 | 0.0000 | 0.0000 | 0.0000 | 0.0000 |
| **2** | 1.0000 |  | 1.0000 | 1.0000 | 1.0000 | 0.9537 | 0.9363 | 0.9100 | 0.9677 | 0.9587 | 0.0000 | 0.0000 | 0.0000 | 0.0000 | 0.0000 | 0.0000 | 0.0000 | 0.0000 | 0.0000 | 0.0000 | 0.0000 | 0.0000 | 0.0000 | 0.0000 | 0.0000 | 0.0000 | 0.0000 | 0.0000 | 0.0000 |
| **3** | 1.0000 | 1.0000 |  | 1.0000 | 1.0000 | 1.0000 | 1.0000 | 1.0000 | 1.0000 | 1.0000 | 0.0000 | 0.0000 | 0.0000 | 0.0000 | 0.0011 | 0.0003 | 0.0000 | 0.0000 | 0.0000 | 0.0034 | 0.0000 | 0.0000 | 0.0000 | 0.0000 | 0.0027 | 0.0000 | 0.0000 | 0.0000 | 0.0000 |
| **4** | 1.0000 | 1.0000 | 1.0000 |  | 1.0000 | 1.0000 | 1.0000 | 1.0000 | 1.0000 | 1.0000 | 0.0000 | 0.0000 | 0.0000 | 0.0000 | 0.0027 | 0.0008 | 0.0000 | 0.0000 | 0.0000 | 0.0073 | 0.0000 | 0.0000 | 0.0000 | 0.0000 | 0.0059 | 0.0001 | 0.0000 | 0.0000 | 0.0000 |
| **5** | 1.0000 | 1.0000 | 1.0000 | 1.0000 |  | 0.9348 | 0.9126 | 0.8800 | 0.9532 | 0.9413 | 0.0000 | 0.0000 | 0.0000 | 0.0000 | 0.0000 | 0.0000 | 0.0000 | 0.0000 | 0.0000 | 0.0000 | 0.0000 | 0.0000 | 0.0000 | 0.0000 | 0.0000 | 0.0000 | 0.0000 | 0.0000 | 0.0000 |
| **6** | 1.0000 | 0.9537 | 1.0000 | 1.0000 | 0.9348 |  | 1.0000 | 1.0000 | 1.0000 | 1.0000 | 0.0411 | 0.0000 | 0.0000 | 0.0000 | 0.4590 | 0.3023 | 0.0271 | 0.0002 | 0.0000 | 0.6098 | 0.0021 | 0.0000 | 0.0000 | 0.0000 | 0.5774 | 0.1185 | 0.0000 | 0.0000 | 0.0000 |
| **7** | 0.9999 | 0.9363 | 1.0000 | 1.0000 | 0.9126 | 1.0000 |  | 1.0000 | 1.0000 | 1.0000 | 0.0544 | 0.0000 | 0.0000 | 0.0000 | 0.5164 | 0.3533 | 0.0366 | 0.0003 | 0.0000 | 0.6647 | 0.0032 | 0.0000 | 0.0000 | 0.0000 | 0.6336 | 0.1480 | 0.0000 | 0.0000 | 0.0000 |
| **8** | 0.9999 | 0.9100 | 1.0000 | 1.0000 | 0.8800 | 1.0000 | 1.0000 |  | 1.0000 | 1.0000 | 0.0743 | 0.0000 | 0.0000 | 0.0000 | 0.5840 | 0.4173 | 0.0512 | 0.0005 | 0.0000 | 0.7256 | 0.0050 | 0.0000 | 0.0000 | 0.0000 | 0.6967 | 0.1888 | 0.0000 | 0.0000 | 0.0000 |
| **9** | 1.0000 | 0.9677 | 1.0000 | 1.0000 | 0.9532 | 1.0000 | 1.0000 | 1.0000 |  | 1.0000 | 0.0302 | 0.0000 | 0.0000 | 0.0000 | 0.4003 | 0.2531 | 0.0195 | 0.0001 | 0.0000 | 0.5502 | 0.0014 | 0.0000 | 0.0000 | 0.0000 | 0.5173 | 0.0925 | 0.0000 | 0.0000 | 0.0000 |
| **10** | 1.0000 | 0.9587 | 1.0000 | 1.0000 | 0.9413 | 1.0000 | 1.0000 | 1.0000 | 1.0000 |  | 0.0372 | 0.0000 | 0.0000 | 0.0000 | 0.4396 | 0.2857 | 0.0244 | 0.0002 | 0.0000 | 0.5905 | 0.0019 | 0.0000 | 0.0000 | 0.0000 | 0.5579 | 0.1094 | 0.0000 | 0.0000 | 0.0000 |
| **11** | 0.0000 | 0.0000 | 0.0000 | 0.0000 | 0.0000 | 0.0411 | 0.0544 | 0.0743 | 0.0302 | 0.0372 |  | 1.0000 | 0.9744 | 0.9796 | 1.0000 | 1.0000 | 1.0000 | 1.0000 | 1.0000 | 1.0000 | 1.0000 | 1.0000 | 0.9998 | 0.9891 | 1.0000 | 1.0000 | 1.0000 | 0.9999 | 1.0000 |
| **12** | 0.0000 | 0.0000 | 0.0000 | 0.0000 | 0.0000 | 0.0000 | 0.0000 | 0.0000 | 0.0000 | 0.0000 | 1.0000 |  | 1.0000 | 1.0000 | 0.9978 | 0.9996 | 1.0000 | 1.0000 | 1.0000 | 0.9918 | 1.0000 | 1.0000 | 1.0000 | 1.0000 | 0.9937 | 1.0000 | 1.0000 | 1.0000 | 1.0000 |
| **13** | 0.0000 | 0.0000 | 0.0000 | 0.0000 | 0.0000 | 0.0000 | 0.0000 | 0.0000 | 0.0000 | 0.0000 | 0.9744 | 1.0000 |  | 1.0000 | 0.5604 | 0.7192 | 0.9852 | 1.0000 | 1.0000 | 0.4101 | 0.9996 | 1.0000 | 1.0000 | 1.0000 | 0.4421 | 0.9040 | 1.0000 | 1.0000 | 1.0000 |
| **14** | 0.0000 | 0.0000 | 0.0000 | 0.0000 | 0.0000 | 0.0000 | 0.0000 | 0.0000 | 0.0000 | 0.0000 | 0.9796 | 1.0000 | 1.0000 |  | 0.5941 | 0.7480 | 0.9885 | 1.0000 | 1.0000 | 0.4432 | 0.9998 | 1.0000 | 1.0000 | 1.0000 | 0.4757 | 0.9187 | 1.0000 | 1.0000 | 1.0000 |
| **15** | 0.0001 | 0.0000 | 0.0011 | 0.0027 | 0.0000 | 0.4590 | 0.5164 | 0.5840 | 0.4003 | 0.4396 | 1.0000 | 0.9978 | 0.5604 | 0.5941 |  | 1.0000 | 1.0000 | 1.0000 | 0.9967 | 1.0000 | 1.0000 | 0.9991 | 0.9379 | 0.6773 | 1.0000 | 1.0000 | 0.9994 | 0.9518 | 0.9903 |
| **16** | 0.0000 | 0.0000 | 0.0003 | 0.0008 | 0.0000 | 0.3023 | 0.3533 | 0.4173 | 0.2531 | 0.2857 | 1.0000 | 0.9996 | 0.7192 | 0.7480 | 1.0000 |  | 1.0000 | 1.0000 | 0.9994 | 1.0000 | 1.0000 | 0.9999 | 0.9773 | 0.8148 | 1.0000 | 1.0000 | 0.9999 | 0.9834 | 0.9977 |
| **17** | 0.0000 | 0.0000 | 0.0000 | 0.0000 | 0.0000 | 0.0271 | 0.0366 | 0.0512 | 0.0195 | 0.0244 | 1.0000 | 1.0000 | 0.9852 | 0.9885 | 1.0000 | 1.0000 |  | 1.0000 | 1.0000 | 1.0000 | 1.0000 | 1.0000 | 0.9999 | 0.9942 | 1.0000 | 1.0000 | 1.0000 | 1.0000 | 1.0000 |
| **18** | 0.0000 | 0.0000 | 0.0000 | 0.0000 | 0.0000 | 0.0002 | 0.0003 | 0.0005 | 0.0001 | 0.0002 | 1.0000 | 1.0000 | 1.0000 | 1.0000 | 1.0000 | 1.0000 | 1.0000 |  | 1.0000 | 0.9999 | 1.0000 | 1.0000 | 1.0000 | 1.0000 | 0.9999 | 1.0000 | 1.0000 | 1.0000 | 1.0000 |
| **19** | 0.0000 | 0.0000 | 0.0000 | 0.0000 | 0.0000 | 0.0000 | 0.0000 | 0.0000 | 0.0000 | 0.0000 | 1.0000 | 1.0000 | 1.0000 | 1.0000 | 0.9967 | 0.9994 | 1.0000 | 1.0000 |  | 0.9886 | 1.0000 | 1.0000 | 1.0000 | 1.0000 | 0.9912 | 1.0000 | 1.0000 | 1.0000 | 1.0000 |
| **20** | 0.0005 | 0.0000 | 0.0034 | 0.0073 | 0.0000 | 0.6098 | 0.6647 | 0.7256 | 0.5502 | 0.5905 | 1.0000 | 0.9918 | 0.4101 | 0.4432 | 1.0000 | 1.0000 | 1.0000 | 0.9999 | 0.9886 |  | 1.0000 | 0.9960 | 0.8707 | 0.5300 | 1.0000 | 1.0000 | 0.9971 | 0.8947 | 0.9718 |
| **21** | 0.0000 | 0.0000 | 0.0000 | 0.0000 | 0.0000 | 0.0021 | 0.0032 | 0.0050 | 0.0014 | 0.0019 | 1.0000 | 1.0000 | 0.9996 | 0.9998 | 1.0000 | 1.0000 | 1.0000 | 1.0000 | 1.0000 | 1.0000 |  | 1.0000 | 1.0000 | 0.9999 | 1.0000 | 1.0000 | 1.0000 | 1.0000 | 1.0000 |
| **22** | 0.0000 | 0.0000 | 0.0000 | 0.0000 | 0.0000 | 0.0000 | 0.0000 | 0.0000 | 0.0000 | 0.0000 | 1.0000 | 1.0000 | 1.0000 | 1.0000 | 0.9991 | 0.9999 | 1.0000 | 1.0000 | 1.0000 | 0.9960 | 1.0000 |  | 1.0000 | 1.0000 | 0.9970 | 1.0000 | 1.0000 | 1.0000 | 1.0000 |
| **23** | 0.0000 | 0.0000 | 0.0000 | 0.0000 | 0.0000 | 0.0000 | 0.0000 | 0.0000 | 0.0000 | 0.0000 | 0.9998 | 1.0000 | 1.0000 | 1.0000 | 0.9379 | 0.9773 | 0.9999 | 1.0000 | 1.0000 | 0.8707 | 1.0000 | 1.0000 |  | 1.0000 | 0.8883 | 0.9974 | 1.0000 | 1.0000 | 1.0000 |
| **24** | 0.0000 | 0.0000 | 0.0000 | 0.0000 | 0.0000 | 0.0000 | 0.0000 | 0.0000 | 0.0000 | 0.0000 | 0.9891 | 1.0000 | 1.0000 | 1.0000 | 0.6773 | 0.8148 | 0.9942 | 1.0000 | 1.0000 | 0.5300 | 0.9999 | 1.0000 | 1.0000 |  | 0.5629 | 0.9488 | 1.0000 | 1.0000 | 1.0000 |
| **25** | 0.0004 | 0.0000 | 0.0027 | 0.0059 | 0.0000 | 0.5774 | 0.6336 | 0.6967 | 0.5173 | 0.5579 | 1.0000 | 0.9937 | 0.4421 | 0.4757 | 1.0000 | 1.0000 | 1.0000 | 0.9999 | 0.9912 | 1.0000 | 1.0000 | 0.9970 | 0.8883 | 0.5629 |  | 1.0000 | 0.9979 | 0.9099 | 0.9773 |
| **26** | 0.0000 | 0.0000 | 0.0000 | 0.0001 | 0.0000 | 0.1185 | 0.1480 | 0.1888 | 0.0925 | 0.1094 | 1.0000 | 1.0000 | 0.9040 | 0.9187 | 1.0000 | 1.0000 | 1.0000 | 1.0000 | 1.0000 | 1.0000 | 1.0000 | 1.0000 | 0.9974 | 0.9488 | 1.0000 |  | 1.0000 | 0.9983 | 0.9999 |
| **27** | 0.0000 | 0.0000 | 0.0000 | 0.0000 | 0.0000 | 0.0000 | 0.0000 | 0.0000 | 0.0000 | 0.0000 | 1.0000 | 1.0000 | 1.0000 | 1.0000 | 0.9994 | 0.9999 | 1.0000 | 1.0000 | 1.0000 | 0.9971 | 1.0000 | 1.0000 | 1.0000 | 1.0000 | 0.9979 | 1.0000 |  | 1.0000 | 1.0000 |
| **28** | 0.0000 | 0.0000 | 0.0000 | 0.0000 | 0.0000 | 0.0000 | 0.0000 | 0.0000 | 0.0000 | 0.0000 | 0.9999 | 1.0000 | 1.0000 | 1.0000 | 0.9518 | 0.9834 | 1.0000 | 1.0000 | 1.0000 | 0.8947 | 1.0000 | 1.0000 | 1.0000 | 1.0000 | 0.9099 | 0.9983 | 1.0000 |  | 1.0000 |
| **29** | 0.0000 | 0.0000 | 0.0000 | 0.0000 | 0.0000 | 0.0000 | 0.0000 | 0.0000 | 0.0000 | 0.0000 | 1.0000 | 1.0000 | 1.0000 | 1.0000 | 0.9903 | 0.9977 | 1.0000 | 1.0000 | 1.0000 | 0.9718 | 1.0000 | 1.0000 | 1.0000 | 1.0000 | 0.9773 | 0.9999 | 1.0000 | 1.0000 |  |

**Supplementary Table 2A***. post hoc* Scheffé test (p<0.05 marked in red) for daily activity counts over time (days) over the 24 hours.

**Supplementary Table 2B***. post hoc* Scheffé test (p<0.05 marked in red) for daily activity counts over time (days) during lights off.

| **Day** | **1** | **2** | **3** | **4** | **5** | **6** | **7** | **8** | **9** | **10** | **11** | **12** | **13** | **14** | **15** | **16** | **17** | **18** | **19** | **20** | **21** | **22** | **23** | **24** | **25** | **26** | **27** | **28** | **29** |
| --- | --- | --- | --- | --- | --- | --- | --- | --- | --- | --- | --- | --- | --- | --- | --- | --- | --- | --- | --- | --- | --- | --- | --- | --- | --- | --- | --- | --- | --- |
| **1** |  | 1.0000 | 1.0000 | 1.0000 | 1.0000 | 1.0000 | 1.0000 | 1.0000 | 1.0000 | 1.0000 | 0.1609 | 0.0000 | 0.0000 | 0.0000 | 0.0000 | 0.0502 | 0.0000 | 0.0025 | 0.0001 | 0.0000 | 0.0726 | 0.0002 | 0.0000 | 0.0000 | 0.0000 | 0.7310 | 0.0028 | 0.0000 | 0.0000 |
| **2** | 1.0000 |  | 1.0000 | 1.0000 | 1.0000 | 0.9991 | 0.9985 | 0.8443 | 0.9983 | 0.9988 | 0.0001 | 0.0000 | 0.0000 | 0.0000 | 0.0000 | 0.0000 | 0.0000 | 0.0000 | 0.0000 | 0.0000 | 0.0000 | 0.0000 | 0.0000 | 0.0000 | 0.0000 | 0.0129 | 0.0000 | 0.0000 | 0.0000 |
| **3** | 1.0000 | 1.0000 |  | 1.0000 | 1.0000 | 1.0000 | 1.0000 | 0.9998 | 1.0000 | 1.0000 | 0.0451 | 0.0000 | 0.0000 | 0.0000 | 0.0000 | 0.0099 | 0.0000 | 0.0003 | 0.0000 | 0.0000 | 0.0158 | 0.0000 | 0.0000 | 0.0000 | 0.0000 | 0.4454 | 0.0003 | 0.0000 | 0.0000 |
| **4** | 1.0000 | 1.0000 | 1.0000 |  | 1.0000 | 1.0000 | 1.0000 | 0.9998 | 1.0000 | 1.0000 | 0.0520 | 0.0000 | 0.0000 | 0.0000 | 0.0000 | 0.0118 | 0.0000 | 0.0003 | 0.0000 | 0.0000 | 0.0187 | 0.0000 | 0.0000 | 0.0000 | 0.0000 | 0.4745 | 0.0004 | 0.0000 | 0.0000 |
| **5** | 1.0000 | 1.0000 | 1.0000 | 1.0000 |  | 1.0000 | 1.0000 | 1.0000 | 1.0000 | 1.0000 | 0.1861 | 0.0000 | 0.0000 | 0.0000 | 0.0000 | 0.0609 | 0.0001 | 0.0034 | 0.0001 | 0.0000 | 0.0869 | 0.0002 | 0.0000 | 0.0000 | 0.0000 | 0.7649 | 0.0037 | 0.0000 | 0.0000 |
| **6** | 1.0000 | 0.9991 | 1.0000 | 1.0000 | 1.0000 |  | 1.0000 | 1.0000 | 1.0000 | 1.0000 | 0.7174 | 0.0005 | 0.0000 | 0.0000 | 0.0000 | 0.4514 | 0.0055 | 0.0891 | 0.0106 | 0.0001 | 0.5304 | 0.0142 | 0.0031 | 0.0003 | 0.0000 | 0.9900 | 0.0946 | 0.0012 | 0.0029 |
| **7** | 1.0000 | 0.9985 | 1.0000 | 1.0000 | 1.0000 | 1.0000 |  | 1.0000 | 1.0000 | 1.0000 | 0.7591 | 0.0007 | 0.0000 | 0.0000 | 0.0000 | 0.5007 | 0.0076 | 0.1095 | 0.0142 | 0.0002 | 0.5797 | 0.0189 | 0.0044 | 0.0005 | 0.0000 | 0.9933 | 0.1160 | 0.0018 | 0.0041 |
| **8** | 1.0000 | 0.8443 | 0.9998 | 0.9998 | 1.0000 | 1.0000 | 1.0000 |  | 1.0000 | 1.0000 | 0.9955 | 0.0533 | 0.0011 | 0.0078 | 0.0017 | 0.9664 | 0.2130 | 0.6866 | 0.2929 | 0.0204 | 0.9799 | 0.3362 | 0.1584 | 0.0439 | 0.0076 | 1.0000 | 0.6993 | 0.0942 | 0.1520 |
| **9** | 1.0000 | 0.9983 | 1.0000 | 1.0000 | 1.0000 | 1.0000 | 1.0000 | 1.0000 |  | 1.0000 | 0.7688 | 0.0008 | 0.0000 | 0.0000 | 0.0000 | 0.5129 | 0.0082 | 0.1151 | 0.0153 | 0.0002 | 0.5917 | 0.0203 | 0.0048 | 0.0006 | 0.0000 | 0.9939 | 0.1217 | 0.0019 | 0.0044 |
| **10** | 1.0000 | 0.9988 | 1.0000 | 1.0000 | 1.0000 | 1.0000 | 1.0000 | 1.0000 | 1.0000 |  | 0.7397 | 0.0006 | 0.0000 | 0.0000 | 0.0000 | 0.4772 | 0.0066 | 0.0994 | 0.0124 | 0.0001 | 0.5564 | 0.0166 | 0.0038 | 0.0004 | 0.0000 | 0.9919 | 0.1054 | 0.0015 | 0.0035 |
| **11** | 0.1609 | 0.0001 | 0.0451 | 0.0520 | 0.1861 | 0.7174 | 0.7591 | 0.9955 | 0.7688 | 0.7397 |  | 0.9999 | 0.9610 | 0.9949 | 0.9728 | 1.0000 | 1.0000 | 1.0000 | 1.0000 | 0.9989 | 1.0000 | 1.0000 | 1.0000 | 0.9998 | 0.9947 | 1.0000 | 1.0000 | 1.0000 | 1.0000 |
| **12** | 0.0000 | 0.0000 | 0.0000 | 0.0000 | 0.0000 | 0.0005 | 0.0007 | 0.0533 | 0.0008 | 0.0006 | 0.9999 |  | 1.0000 | 1.0000 | 1.0000 | 1.0000 | 1.0000 | 1.0000 | 1.0000 | 1.0000 | 1.0000 | 1.0000 | 1.0000 | 1.0000 | 1.0000 | 0.9578 | 1.0000 | 1.0000 | 1.0000 |
| **13** | 0.0000 | 0.0000 | 0.0000 | 0.0000 | 0.0000 | 0.0000 | 0.0000 | 0.0011 | 0.0000 | 0.0000 | 0.9610 | 1.0000 |  | 1.0000 | 1.0000 | 0.9945 | 1.0000 | 1.0000 | 1.0000 | 1.0000 | 0.9897 | 1.0000 | 1.0000 | 1.0000 | 1.0000 | 0.5257 | 1.0000 | 1.0000 | 1.0000 |
| **14** | 0.0000 | 0.0000 | 0.0000 | 0.0000 | 0.0000 | 0.0000 | 0.0000 | 0.0078 | 0.0000 | 0.0000 | 0.9949 | 1.0000 | 1.0000 |  | 1.0000 | 0.9997 | 1.0000 | 1.0000 | 1.0000 | 1.0000 | 0.9992 | 1.0000 | 1.0000 | 1.0000 | 1.0000 | 0.7858 | 1.0000 | 1.0000 | 1.0000 |
| **15** | 0.0000 | 0.0000 | 0.0000 | 0.0000 | 0.0000 | 0.0000 | 0.0000 | 0.0017 | 0.0000 | 0.0000 | 0.9728 | 1.0000 | 1.0000 | 1.0000 |  | 0.9967 | 1.0000 | 1.0000 | 1.0000 | 1.0000 | 0.9934 | 1.0000 | 1.0000 | 1.0000 | 1.0000 | 0.5833 | 1.0000 | 1.0000 | 1.0000 |
| **16** | 0.0502 | 0.0000 | 0.0099 | 0.0118 | 0.0609 | 0.4514 | 0.5007 | 0.9664 | 0.5129 | 0.4772 | 1.0000 | 1.0000 | 0.9945 | 0.9997 | 0.9967 |  | 1.0000 | 1.0000 | 1.0000 | 1.0000 | 1.0000 | 1.0000 | 1.0000 | 1.0000 | 0.9996 | 1.0000 | 1.0000 | 1.0000 | 1.0000 |
| **17** | 0.0000 | 0.0000 | 0.0000 | 0.0000 | 0.0001 | 0.0055 | 0.0076 | 0.2130 | 0.0082 | 0.0066 | 1.0000 | 1.0000 | 1.0000 | 1.0000 | 1.0000 | 1.0000 |  | 1.0000 | 1.0000 | 1.0000 | 1.0000 | 1.0000 | 1.0000 | 1.0000 | 1.0000 | 0.9966 | 1.0000 | 1.0000 | 1.0000 |
| **18** | 0.0025 | 0.0000 | 0.0003 | 0.0003 | 0.0034 | 0.0891 | 0.1095 | 0.6866 | 0.1151 | 0.0994 | 1.0000 | 1.0000 | 1.0000 | 1.0000 | 1.0000 | 1.0000 | 1.0000 |  | 1.0000 | 1.0000 | 1.0000 | 1.0000 | 1.0000 | 1.0000 | 1.0000 | 1.0000 | 1.0000 | 1.0000 | 1.0000 |
| **19** | 0.0001 | 0.0000 | 0.0000 | 0.0000 | 0.0001 | 0.0106 | 0.0142 | 0.2929 | 0.0153 | 0.0124 | 1.0000 | 1.0000 | 1.0000 | 1.0000 | 1.0000 | 1.0000 | 1.0000 | 1.0000 |  | 1.0000 | 1.0000 | 1.0000 | 1.0000 | 1.0000 | 1.0000 | 0.9987 | 1.0000 | 1.0000 | 1.0000 |
| **20** | 0.0000 | 0.0000 | 0.0000 | 0.0000 | 0.0000 | 0.0001 | 0.0002 | 0.0204 | 0.0002 | 0.0001 | 0.9989 | 1.0000 | 1.0000 | 1.0000 | 1.0000 | 1.0000 | 1.0000 | 1.0000 | 1.0000 |  | 0.9999 | 1.0000 | 1.0000 | 1.0000 | 1.0000 | 0.8891 | 1.0000 | 1.0000 | 1.0000 |
| **21** | 0.0726 | 0.0000 | 0.0158 | 0.0187 | 0.0869 | 0.5304 | 0.5797 | 0.9799 | 0.5917 | 0.5564 | 1.0000 | 1.0000 | 0.9897 | 0.9992 | 0.9934 | 1.0000 | 1.0000 | 1.0000 | 1.0000 | 0.9999 |  | 1.0000 | 1.0000 | 1.0000 | 0.9992 | 1.0000 | 1.0000 | 1.0000 | 1.0000 |
| **22** | 0.0002 | 0.0000 | 0.0000 | 0.0000 | 0.0002 | 0.0142 | 0.0189 | 0.3362 | 0.0203 | 0.0166 | 1.0000 | 1.0000 | 1.0000 | 1.0000 | 1.0000 | 1.0000 | 1.0000 | 1.0000 | 1.0000 | 1.0000 | 1.0000 |  | 1.0000 | 1.0000 | 1.0000 | 0.9992 | 1.0000 | 1.0000 | 1.0000 |
| **23** | 0.0000 | 0.0000 | 0.0000 | 0.0000 | 0.0000 | 0.0031 | 0.0044 | 0.1584 | 0.0048 | 0.0038 | 1.0000 | 1.0000 | 1.0000 | 1.0000 | 1.0000 | 1.0000 | 1.0000 | 1.0000 | 1.0000 | 1.0000 | 1.0000 | 1.0000 |  | 1.0000 | 1.0000 | 0.9930 | 1.0000 | 1.0000 | 1.0000 |
| **24** | 0.0000 | 0.0000 | 0.0000 | 0.0000 | 0.0000 | 0.0003 | 0.0005 | 0.0439 | 0.0006 | 0.0004 | 0.9998 | 1.0000 | 1.0000 | 1.0000 | 1.0000 | 1.0000 | 1.0000 | 1.0000 | 1.0000 | 1.0000 | 1.0000 | 1.0000 | 1.0000 |  | 1.0000 | 0.9470 | 1.0000 | 1.0000 | 1.0000 |
| **25** | 0.0000 | 0.0000 | 0.0000 | 0.0000 | 0.0000 | 0.0000 | 0.0000 | 0.0076 | 0.0000 | 0.0000 | 0.9947 | 1.0000 | 1.0000 | 1.0000 | 1.0000 | 0.9996 | 1.0000 | 1.0000 | 1.0000 | 1.0000 | 0.9992 | 1.0000 | 1.0000 | 1.0000 |  | 0.7827 | 1.0000 | 1.0000 | 1.0000 |
| **26** | 0.7310 | 0.0129 | 0.4454 | 0.4745 | 0.7649 | 0.9900 | 0.9933 | 1.0000 | 0.9939 | 0.9919 | 1.0000 | 0.9578 | 0.5257 | 0.7858 | 0.5833 | 1.0000 | 0.9966 | 1.0000 | 0.9987 | 0.8891 | 1.0000 | 0.9992 | 0.9930 | 0.9470 | 0.7827 |  | 1.0000 | 0.9812 | 0.9923 |
| **27** | 0.0028 | 0.0000 | 0.0003 | 0.0004 | 0.0037 | 0.0946 | 0.1160 | 0.6993 | 0.1217 | 0.1054 | 1.0000 | 1.0000 | 1.0000 | 1.0000 | 1.0000 | 1.0000 | 1.0000 | 1.0000 | 1.0000 | 1.0000 | 1.0000 | 1.0000 | 1.0000 | 1.0000 | 1.0000 | 1.0000 |  | 1.0000 | 1.0000 |
| **28** | 0.0000 | 0.0000 | 0.0000 | 0.0000 | 0.0000 | 0.0012 | 0.0018 | 0.0942 | 0.0019 | 0.0015 | 1.0000 | 1.0000 | 1.0000 | 1.0000 | 1.0000 | 1.0000 | 1.0000 | 1.0000 | 1.0000 | 1.0000 | 1.0000 | 1.0000 | 1.0000 | 1.0000 | 1.0000 | 0.9812 | 1.0000 |  | 1.0000 |
| **29** | 0.0000 | 0.0000 | 0.0000 | 0.0000 | 0.0000 | 0.0029 | 0.0041 | 0.1520 | 0.0044 | 0.0035 | 1.0000 | 1.0000 | 1.0000 | 1.0000 | 1.0000 | 1.0000 | 1.0000 | 1.0000 | 1.0000 | 1.0000 | 1.0000 | 1.0000 | 1.0000 | 1.0000 | 1.0000 | 0.9923 | 1.0000 | 1.0000 |  |

**Supplementary Table 2C** *post hoc* Scheffé test (p<0.05 marked in red) for daily activity counts over time (days) during lights on.

| **Day** | **1** | **2** | **3** | **4** | **5** | **6** | **7** | **8** | **9** | **10** | **11** | **12** | **13** | **14** | **15** | **16** | **17** | **18** | **19** | **20** | **21** | **22** | **23** | **24** | **25** | **26** | **27** | **28** | **29** |
| --- | --- | --- | --- | --- | --- | --- | --- | --- | --- | --- | --- | --- | --- | --- | --- | --- | --- | --- | --- | --- | --- | --- | --- | --- | --- | --- | --- | --- | --- |
| **1** |  | 1.0000 | 1.0000 | 1.0000 | 1.0000 | 0.9997 | 0.9996 | 1.0000 | 0.9999 | 0.9998 | 0.0000 | 0.0000 | 0.0000 | 0.0000 | 0.8677 | 0.0037 | 0.0014 | 0.0000 | 0.0000 | 0.8352 | 0.0000 | 0.0000 | 0.0000 | 0.0000 | 0.8760 | 0.0000 | 0.0000 | 0.0000 | 0.0000 |
| **2** | 1.0000 |  | 1.0000 | 1.0000 | 1.0000 | 0.9944 | 0.9917 | 0.9998 | 0.9982 | 0.9962 | 0.0000 | 0.0000 | 0.0000 | 0.0000 | 0.6221 | 0.0004 | 0.0001 | 0.0000 | 0.0000 | 0.5699 | 0.0000 | 0.0000 | 0.0000 | 0.0000 | 0.6363 | 0.0000 | 0.0000 | 0.0000 | 0.0000 |
| **3** | 1.0000 | 1.0000 |  | 1.0000 | 0.9992 | 1.0000 | 1.0000 | 1.0000 | 1.0000 | 1.0000 | 0.0003 | 0.0000 | 0.0000 | 0.0000 | 0.9963 | 0.0834 | 0.0450 | 0.0000 | 0.0000 | 0.9942 | 0.0000 | 0.0000 | 0.0000 | 0.0000 | 0.9968 | 0.0001 | 0.0000 | 0.0000 | 0.0000 |
| **4** | 1.0000 | 1.0000 | 1.0000 |  | 0.9967 | 1.0000 | 1.0000 | 1.0000 | 1.0000 | 1.0000 | 0.0009 | 0.0000 | 0.0000 | 0.0000 | 0.9991 | 0.1509 | 0.0884 | 0.0000 | 0.0000 | 0.9985 | 0.0000 | 0.0000 | 0.0000 | 0.0000 | 0.9992 | 0.0004 | 0.0000 | 0.0000 | 0.0000 |
| **5** | 1.0000 | 1.0000 | 0.9992 | 0.9967 |  | 0.5254 | 0.4785 | 0.8215 | 0.6500 | 0.5708 | 0.0000 | 0.0000 | 0.0000 | 0.0000 | 0.0279 | 0.0000 | 0.0000 | 0.0000 | 0.0000 | 0.0209 | 0.0000 | 0.0000 | 0.0000 | 0.0000 | 0.0301 | 0.0000 | 0.0000 | 0.0000 | 0.0000 |
| **6** | 0.9997 | 0.9944 | 1.0000 | 1.0000 | 0.5254 |  | 1.0000 | 1.0000 | 1.0000 | 1.0000 | 0.1969 | 0.0128 | 0.0000 | 0.0000 | 1.0000 | 0.9273 | 0.8640 | 0.0120 | 0.0011 | 1.0000 | 0.0156 | 0.0032 | 0.0000 | 0.0000 | 1.0000 | 0.1315 | 0.0007 | 0.0001 | 0.0008 |
| **7** | 0.9996 | 0.9917 | 1.0000 | 1.0000 | 0.4785 | 1.0000 |  | 1.0000 | 1.0000 | 1.0000 | 0.2299 | 0.0168 | 0.0000 | 0.0000 | 1.0000 | 0.9434 | 0.8895 | 0.0159 | 0.0016 | 1.0000 | 0.0204 | 0.0044 | 0.0000 | 0.0000 | 1.0000 | 0.1570 | 0.0010 | 0.0001 | 0.0011 |
| **8** | 1.0000 | 0.9998 | 1.0000 | 1.0000 | 0.8215 | 1.0000 | 1.0000 |  | 1.0000 | 1.0000 | 0.0499 | 0.0014 | 0.0000 | 0.0000 | 1.0000 | 0.7172 | 0.5923 | 0.0013 | 0.0001 | 1.0000 | 0.0018 | 0.0003 | 0.0000 | 0.0000 | 1.0000 | 0.0284 | 0.0000 | 0.0000 | 0.0000 |
| **9** | 0.9999 | 0.9982 | 1.0000 | 1.0000 | 0.6500 | 1.0000 | 1.0000 | 1.0000 |  | 1.0000 | 0.1236 | 0.0058 | 0.0000 | 0.0000 | 1.0000 | 0.8677 | 0.7779 | 0.0055 | 0.0004 | 1.0000 | 0.0073 | 0.0013 | 0.0000 | 0.0000 | 1.0000 | 0.0777 | 0.0003 | 0.0000 | 0.0003 |
| **10** | 0.9998 | 0.9962 | 1.0000 | 1.0000 | 0.5708 | 1.0000 | 1.0000 | 1.0000 | 1.0000 |  | 0.1679 | 0.0097 | 0.0000 | 0.0000 | 1.0000 | 0.9088 | 0.8361 | 0.0091 | 0.0008 | 1.0000 | 0.0120 | 0.0024 | 0.0000 | 0.0000 | 1.0000 | 0.1098 | 0.0005 | 0.0001 | 0.0005 |
| **11** | 0.0000 | 0.0000 | 0.0003 | 0.0009 | 0.0000 | 0.1969 | 0.2299 | 0.0499 | 0.1236 | 0.1679 |  | 1.0000 | 1.0000 | 0.9997 | 0.8897 | 1.0000 | 1.0000 | 1.0000 | 1.0000 | 0.9143 | 1.0000 | 1.0000 | 1.0000 | 0.9993 | 0.8821 | 1.0000 | 1.0000 | 1.0000 | 1.0000 |
| **12** | 0.0000 | 0.0000 | 0.0000 | 0.0000 | 0.0000 | 0.0128 | 0.0168 | 0.0014 | 0.0058 | 0.0097 | 1.0000 |  | 1.0000 | 1.0000 | 0.3893 | 1.0000 | 1.0000 | 1.0000 | 1.0000 | 0.4408 | 1.0000 | 1.0000 | 1.0000 | 1.0000 | 0.3754 | 1.0000 | 1.0000 | 1.0000 | 1.0000 |
| **13** | 0.0000 | 0.0000 | 0.0000 | 0.0000 | 0.0000 | 0.0000 | 0.0000 | 0.0000 | 0.0000 | 0.0000 | 1.0000 | 1.0000 |  | 1.0000 | 0.0055 | 0.8991 | 0.9493 | 1.0000 | 1.0000 | 0.0077 | 1.0000 | 1.0000 | 1.0000 | 1.0000 | 0.0050 | 1.0000 | 1.0000 | 1.0000 | 1.0000 |
| **14** | 0.0000 | 0.0000 | 0.0000 | 0.0000 | 0.0000 | 0.0000 | 0.0000 | 0.0000 | 0.0000 | 0.0000 | 0.9997 | 1.0000 | 1.0000 |  | 0.0020 | 0.8125 | 0.8926 | 1.0000 | 1.0000 | 0.0029 | 1.0000 | 1.0000 | 1.0000 | 1.0000 | 0.0018 | 0.9999 | 1.0000 | 1.0000 | 1.0000 |
| **15** | 0.8677 | 0.6221 | 0.9963 | 0.9991 | 0.0279 | 1.0000 | 1.0000 | 1.0000 | 1.0000 | 1.0000 | 0.8897 | 0.3893 | 0.0055 | 0.0020 |  | 0.9999 | 0.9997 | 0.3797 | 0.1215 | 1.0000 | 0.4221 | 0.2090 | 0.0126 | 0.0011 | 1.0000 | 0.8240 | 0.0960 | 0.0300 | 0.1002 |
| **16** | 0.0037 | 0.0004 | 0.0834 | 0.1509 | 0.0000 | 0.9273 | 0.9434 | 0.7172 | 0.8677 | 0.9088 | 1.0000 | 1.0000 | 0.8991 | 0.8125 | 0.9999 |  | 1.0000 | 1.0000 | 0.9984 | 1.0000 | 1.0000 | 0.9997 | 0.9494 | 0.7440 | 0.9999 | 1.0000 | 0.9973 | 0.9807 | 0.9975 |
| **17** | 0.0014 | 0.0001 | 0.0450 | 0.0884 | 0.0000 | 0.8640 | 0.8895 | 0.5923 | 0.7779 | 0.8361 | 1.0000 | 1.0000 | 0.9493 | 0.8926 | 0.9997 | 1.0000 |  | 1.0000 | 0.9996 | 0.9999 | 1.0000 | 0.9999 | 0.9777 | 0.8424 | 0.9997 | 1.0000 | 0.9993 | 0.9928 | 0.9993 |
| **18** | 0.0000 | 0.0000 | 0.0000 | 0.0000 | 0.0000 | 0.0120 | 0.0159 | 0.0013 | 0.0055 | 0.0091 | 1.0000 | 1.0000 | 1.0000 | 1.0000 | 0.3797 | 1.0000 | 1.0000 |  | 1.0000 | 0.4308 | 1.0000 | 1.0000 | 1.0000 | 1.0000 | 0.3658 | 1.0000 | 1.0000 | 1.0000 | 1.0000 |
| **19** | 0.0000 | 0.0000 | 0.0000 | 0.0000 | 0.0000 | 0.0011 | 0.0016 | 0.0001 | 0.0004 | 0.0008 | 1.0000 | 1.0000 | 1.0000 | 1.0000 | 0.1215 | 0.9984 | 0.9996 | 1.0000 |  | 0.1491 | 1.0000 | 1.0000 | 1.0000 | 1.0000 | 0.1145 | 1.0000 | 1.0000 | 1.0000 | 1.0000 |
| **20** | 0.8352 | 0.5699 | 0.9942 | 0.9985 | 0.0209 | 1.0000 | 1.0000 | 1.0000 | 1.0000 | 1.0000 | 0.9143 | 0.4408 | 0.0077 | 0.0029 | 1.0000 | 1.0000 | 0.9999 | 0.4308 | 0.1491 |  | 0.4745 | 0.2480 | 0.0172 | 0.0016 | 1.0000 | 0.8579 | 0.1194 | 0.0395 | 0.1243 |
| **21** | 0.0000 | 0.0000 | 0.0000 | 0.0000 | 0.0000 | 0.0156 | 0.0204 | 0.0018 | 0.0073 | 0.0120 | 1.0000 | 1.0000 | 1.0000 | 1.0000 | 0.4221 | 1.0000 | 1.0000 | 1.0000 | 1.0000 | 0.4745 |  | 1.0000 | 1.0000 | 1.0000 | 0.4078 | 1.0000 | 1.0000 | 1.0000 | 1.0000 |
| **22** | 0.0000 | 0.0000 | 0.0000 | 0.0000 | 0.0000 | 0.0032 | 0.0044 | 0.0003 | 0.0013 | 0.0024 | 1.0000 | 1.0000 | 1.0000 | 1.0000 | 0.2090 | 0.9997 | 0.9999 | 1.0000 | 1.0000 | 0.2480 | 1.0000 |  | 1.0000 | 1.0000 | 0.1989 | 1.0000 | 1.0000 | 1.0000 | 1.0000 |
| **23** | 0.0000 | 0.0000 | 0.0000 | 0.0000 | 0.0000 | 0.0000 | 0.0000 | 0.0000 | 0.0000 | 0.0000 | 1.0000 | 1.0000 | 1.0000 | 1.0000 | 0.0126 | 0.9494 | 0.9777 | 1.0000 | 1.0000 | 0.0172 | 1.0000 | 1.0000 |  | 1.0000 | 0.0116 | 1.0000 | 1.0000 | 1.0000 | 1.0000 |
| **24** | 0.0000 | 0.0000 | 0.0000 | 0.0000 | 0.0000 | 0.0000 | 0.0000 | 0.0000 | 0.0000 | 0.0000 | 0.9993 | 1.0000 | 1.0000 | 1.0000 | 0.0011 | 0.7440 | 0.8424 | 1.0000 | 1.0000 | 0.0016 | 1.0000 | 1.0000 | 1.0000 |  | 0.0009 | 0.9998 | 1.0000 | 1.0000 | 1.0000 |
| **25** | 0.8760 | 0.6363 | 0.9968 | 0.9992 | 0.0301 | 1.0000 | 1.0000 | 1.0000 | 1.0000 | 1.0000 | 0.8821 | 0.3754 | 0.0050 | 0.0018 | 1.0000 | 0.9999 | 0.9997 | 0.3658 | 0.1145 | 1.0000 | 0.4078 | 0.1989 | 0.0116 | 0.0009 |  | 0.8137 | 0.0902 | 0.0278 | 0.0942 |
| **26** | 0.0000 | 0.0000 | 0.0001 | 0.0004 | 0.0000 | 0.1315 | 0.1570 | 0.0284 | 0.0777 | 0.1098 | 1.0000 | 1.0000 | 1.0000 | 0.9999 | 0.8240 | 1.0000 | 1.0000 | 1.0000 | 1.0000 | 0.8579 | 1.0000 | 1.0000 | 1.0000 | 0.9998 | 0.8137 |  | 1.0000 | 1.0000 | 1.0000 |
| **27** | 0.0000 | 0.0000 | 0.0000 | 0.0000 | 0.0000 | 0.0007 | 0.0010 | 0.0000 | 0.0003 | 0.0005 | 1.0000 | 1.0000 | 1.0000 | 1.0000 | 0.0960 | 0.9973 | 0.9993 | 1.0000 | 1.0000 | 0.1194 | 1.0000 | 1.0000 | 1.0000 | 1.0000 | 0.0902 | 1.0000 |  | 1.0000 | 1.0000 |
| **28** | 0.0000 | 0.0000 | 0.0000 | 0.0000 | 0.0000 | 0.0001 | 0.0001 | 0.0000 | 0.0000 | 0.0001 | 1.0000 | 1.0000 | 1.0000 | 1.0000 | 0.0300 | 0.9807 | 0.9928 | 1.0000 | 1.0000 | 0.0395 | 1.0000 | 1.0000 | 1.0000 | 1.0000 | 0.0278 | 1.0000 | 1.0000 |  | 1.0000 |
| **29** | 0.0000 | 0.0000 | 0.0000 | 0.0000 | 0.0000 | 0.0008 | 0.0011 | 0.0000 | 0.0003 | 0.0005 | 1.0000 | 1.0000 | 1.0000 | 1.0000 | 0.1002 | 0.9975 | 0.9993 | 1.0000 | 1.0000 | 0.1243 | 1.0000 | 1.0000 | 1.0000 | 1.0000 | 0.0942 | 1.0000 | 1.0000 | 1.0000 |  |

**Supplementary Table 2D***. post hoc* Scheffé test (p<0.05 marked in red) for daily sleep amount over time (days) over the 24 hours.

| **Day** | **1** | **2** | **3** | **4** | **5** | **6** | **7** | **8** | **9** | **10** | **11** | **12** | **13** | **14** | **15** | **16** | **17** | **18** | **19** | **20** | **21** | **22** | **23** | **24** | **25** | **26** | **27** | **28** | **29** |
| --- | --- | --- | --- | --- | --- | --- | --- | --- | --- | --- | --- | --- | --- | --- | --- | --- | --- | --- | --- | --- | --- | --- | --- | --- | --- | --- | --- | --- | --- |
| **1** |  | 1.0000 | 1.0000 | 1.0000 | 1.0000 | 0.8543 | 0.8713 | 0.8139 | 0.7914 | 1.0000 | 0.0000 | 0.0000 | 0.0000 | 0.0000 | 0.0000 | 0.0000 | 0.0000 | 0.0000 | 0.0000 | 0.0000 | 0.0000 | 0.0000 | 0.0000 | 0.0000 | 0.0000 | 0.0000 | 0.0000 | 0.0000 | 0.0000 |
| **2** | 1.0000 |  | 1.0000 | 1.0000 | 1.0000 | 0.9646 | 0.9705 | 0.9492 | 0.9398 | 1.0000 | 0.0000 | 0.0000 | 0.0000 | 0.0000 | 0.0002 | 0.0000 | 0.0000 | 0.0000 | 0.0000 | 0.0000 | 0.0000 | 0.0000 | 0.0000 | 0.0000 | 0.0000 | 0.0000 | 0.0000 | 0.0000 | 0.0000 |
| **3** | 1.0000 | 1.0000 |  | 1.0000 | 1.0000 | 0.9050 | 0.9177 | 0.8740 | 0.8562 | 1.0000 | 0.0000 | 0.0000 | 0.0000 | 0.0000 | 0.0000 | 0.0000 | 0.0000 | 0.0000 | 0.0000 | 0.0000 | 0.0000 | 0.0000 | 0.0000 | 0.0000 | 0.0000 | 0.0000 | 0.0000 | 0.0000 | 0.0000 |
| **4** | 1.0000 | 1.0000 | 1.0000 |  | 1.0000 | 0.9723 | 0.9771 | 0.9595 | 0.9515 | 1.0000 | 0.0001 | 0.0000 | 0.0000 | 0.0000 | 0.0002 | 0.0000 | 0.0000 | 0.0000 | 0.0000 | 0.0000 | 0.0000 | 0.0000 | 0.0000 | 0.0000 | 0.0000 | 0.0000 | 0.0000 | 0.0000 | 0.0000 |
| **5** | 1.0000 | 1.0000 | 1.0000 | 1.0000 |  | 0.9723 | 0.9771 | 0.9595 | 0.9515 | 1.0000 | 0.0001 | 0.0000 | 0.0000 | 0.0000 | 0.0002 | 0.0000 | 0.0000 | 0.0000 | 0.0000 | 0.0000 | 0.0000 | 0.0000 | 0.0000 | 0.0000 | 0.0000 | 0.0000 | 0.0000 | 0.0000 | 0.0000 |
| **6** | 0.8543 | 0.9646 | 0.9050 | 0.9723 | 0.9723 |  | 1.0000 | 1.0000 | 1.0000 | 0.9986 | 0.9260 | 0.0063 | 0.0000 | 0.0014 | 0.9699 | 0.5490 | 0.0035 | 0.0001 | 0.0001 | 0.7727 | 0.0084 | 0.0000 | 0.0000 | 0.0000 | 0.0391 | 0.0000 | 0.0000 | 0.0000 | 0.0000 |
| **7** | 0.8713 | 0.9705 | 0.9177 | 0.9771 | 0.9771 | 1.0000 |  | 1.0000 | 1.0000 | 0.9989 | 0.9143 | 0.0052 | 0.0000 | 0.0011 | 0.9639 | 0.5198 | 0.0028 | 0.0000 | 0.0001 | 0.7491 | 0.0070 | 0.0000 | 0.0000 | 0.0000 | 0.0337 | 0.0000 | 0.0000 | 0.0000 | 0.0000 |
| **8** | 0.8139 | 0.9492 | 0.8740 | 0.9595 | 0.9595 | 1.0000 | 1.0000 |  | 1.0000 | 0.9974 | 0.9468 | 0.0093 | 0.0000 | 0.0022 | 0.9799 | 0.6101 | 0.0052 | 0.0001 | 0.0001 | 0.8184 | 0.0122 | 0.0000 | 0.0000 | 0.0000 | 0.0530 | 0.0000 | 0.0000 | 0.0000 | 0.0000 |
| **9** | 0.7914 | 0.9398 | 0.8562 | 0.9515 | 0.9515 | 1.0000 | 1.0000 | 1.0000 |  | 0.9966 | 0.9554 | 0.0112 | 0.0000 | 0.0027 | 0.9838 | 0.6399 | 0.0064 | 0.0001 | 0.0002 | 0.8390 | 0.0147 | 0.0000 | 0.0000 | 0.0000 | 0.0613 | 0.0000 | 0.0000 | 0.0000 | 0.0000 |
| **10** | 1.0000 | 1.0000 | 1.0000 | 1.0000 | 1.0000 | 0.9986 | 0.9989 | 0.9974 | 0.9966 |  | 0.0012 | 0.0000 | 0.0000 | 0.0000 | 0.0036 | 0.0000 | 0.0000 | 0.0000 | 0.0000 | 0.0001 | 0.0000 | 0.0000 | 0.0000 | 0.0000 | 0.0000 | 0.0000 | 0.0000 | 0.0000 | 0.0000 |
| **11** | 0.0000 | 0.0000 | 0.0000 | 0.0001 | 0.0001 | 0.9260 | 0.9143 | 0.9468 | 0.9554 | 0.0012 |  | 0.9999 | 0.9284 | 0.9988 | 1.0000 | 1.0000 | 0.9997 | 0.9726 | 0.9781 | 1.0000 | 0.9999 | 0.7188 | 0.0833 | 0.1372 | 1.0000 | 0.8722 | 0.2403 | 0.0310 | 0.0888 |
| **12** | 0.0000 | 0.0000 | 0.0000 | 0.0000 | 0.0000 | 0.0063 | 0.0052 | 0.0093 | 0.0112 | 0.0000 | 0.9999 |  | 1.0000 | 1.0000 | 0.9994 | 1.0000 | 1.0000 | 1.0000 | 1.0000 | 1.0000 | 1.0000 | 1.0000 | 0.9975 | 0.9992 | 1.0000 | 1.0000 | 0.9999 | 0.9862 | 0.9978 |
| **13** | 0.0000 | 0.0000 | 0.0000 | 0.0000 | 0.0000 | 0.0000 | 0.0000 | 0.0000 | 0.0000 | 0.0000 | 0.9284 | 1.0000 |  | 1.0000 | 0.8513 | 0.9982 | 1.0000 | 1.0000 | 1.0000 | 0.9859 | 1.0000 | 1.0000 | 1.0000 | 1.0000 | 1.0000 | 1.0000 | 1.0000 | 1.0000 | 1.0000 |
| **14** | 0.0000 | 0.0000 | 0.0000 | 0.0000 | 0.0000 | 0.0014 | 0.0011 | 0.0022 | 0.0027 | 0.0000 | 0.9988 | 1.0000 | 1.0000 |  | 0.9950 | 1.0000 | 1.0000 | 1.0000 | 1.0000 | 0.9999 | 1.0000 | 1.0000 | 0.9997 | 0.9999 | 1.0000 | 1.0000 | 1.0000 | 0.9978 | 0.9998 |
| **15** | 0.0000 | 0.0002 | 0.0000 | 0.0002 | 0.0002 | 0.9699 | 0.9639 | 0.9799 | 0.9838 | 0.0036 | 1.0000 | 0.9994 | 0.8513 | 0.9950 |  | 1.0000 | 0.9985 | 0.9314 | 0.9429 | 1.0000 | 0.9996 | 0.5701 | 0.0398 | 0.0711 | 1.0000 | 0.7650 | 0.1389 | 0.0128 | 0.0428 |
| **16** | 0.0000 | 0.0000 | 0.0000 | 0.0000 | 0.0000 | 0.5490 | 0.5198 | 0.6101 | 0.6399 | 0.0000 | 1.0000 | 1.0000 | 0.9982 | 1.0000 | 1.0000 |  | 1.0000 | 0.9997 | 0.9998 | 1.0000 | 1.0000 | 0.9737 | 0.4389 | 0.5604 | 1.0000 | 0.9946 | 0.7131 | 0.2541 | 0.4533 |
| **17** | 0.0000 | 0.0000 | 0.0000 | 0.0000 | 0.0000 | 0.0035 | 0.0028 | 0.0052 | 0.0064 | 0.0000 | 0.9997 | 1.0000 | 1.0000 | 1.0000 | 0.9985 | 1.0000 |  | 1.0000 | 1.0000 | 1.0000 | 1.0000 | 1.0000 | 0.9990 | 0.9997 | 1.0000 | 1.0000 | 1.0000 | 0.9932 | 0.9991 |
| **18** | 0.0000 | 0.0000 | 0.0000 | 0.0000 | 0.0000 | 0.0001 | 0.0000 | 0.0001 | 0.0001 | 0.0000 | 0.9726 | 1.0000 | 1.0000 | 1.0000 | 0.9314 | 0.9997 | 1.0000 |  | 1.0000 | 0.9963 | 1.0000 | 1.0000 | 1.0000 | 1.0000 | 1.0000 | 1.0000 | 1.0000 | 1.0000 | 1.0000 |
| **19** | 0.0000 | 0.0000 | 0.0000 | 0.0000 | 0.0000 | 0.0001 | 0.0001 | 0.0001 | 0.0002 | 0.0000 | 0.9781 | 1.0000 | 1.0000 | 1.0000 | 0.9429 | 0.9998 | 1.0000 | 1.0000 |  | 0.9972 | 1.0000 | 1.0000 | 1.0000 | 1.0000 | 1.0000 | 1.0000 | 1.0000 | 1.0000 | 1.0000 |
| **20** | 0.0000 | 0.0000 | 0.0000 | 0.0000 | 0.0000 | 0.7727 | 0.7491 | 0.8184 | 0.8390 | 0.0001 | 1.0000 | 1.0000 | 0.9859 | 0.9999 | 1.0000 | 1.0000 | 1.0000 | 0.9963 | 0.9972 |  | 1.0000 | 0.8982 | 0.2258 | 0.3245 | 1.0000 | 0.9680 | 0.4775 | 0.1060 | 0.2367 |
| **21** | 0.0000 | 0.0000 | 0.0000 | 0.0000 | 0.0000 | 0.0084 | 0.0070 | 0.0122 | 0.0147 | 0.0000 | 0.9999 | 1.0000 | 1.0000 | 1.0000 | 0.9996 | 1.0000 | 1.0000 | 1.0000 | 1.0000 | 1.0000 |  | 1.0000 | 0.9962 | 0.9988 | 1.0000 | 1.0000 | 0.9998 | 0.9810 | 0.9967 |
| **22** | 0.0000 | 0.0000 | 0.0000 | 0.0000 | 0.0000 | 0.0000 | 0.0000 | 0.0000 | 0.0000 | 0.0000 | 0.7188 | 1.0000 | 1.0000 | 1.0000 | 0.5701 | 0.9737 | 1.0000 | 1.0000 | 1.0000 | 0.8982 | 1.0000 |  | 1.0000 | 1.0000 | 1.0000 | 1.0000 | 1.0000 | 1.0000 | 1.0000 |
| **23** | 0.0000 | 0.0000 | 0.0000 | 0.0000 | 0.0000 | 0.0000 | 0.0000 | 0.0000 | 0.0000 | 0.0000 | 0.0833 | 0.9975 | 1.0000 | 0.9997 | 0.0398 | 0.4389 | 0.9990 | 1.0000 | 1.0000 | 0.2258 | 0.9962 | 1.0000 |  | 1.0000 | 0.9705 | 1.0000 | 1.0000 | 1.0000 | 1.0000 |
| **24** | 0.0000 | 0.0000 | 0.0000 | 0.0000 | 0.0000 | 0.0000 | 0.0000 | 0.0000 | 0.0000 | 0.0000 | 0.1372 | 0.9992 | 1.0000 | 0.9999 | 0.0711 | 0.5604 | 0.9997 | 1.0000 | 1.0000 | 0.3245 | 0.9988 | 1.0000 | 1.0000 |  | 0.9872 | 1.0000 | 1.0000 | 1.0000 | 1.0000 |
| **25** | 0.0000 | 0.0000 | 0.0000 | 0.0000 | 0.0000 | 0.0391 | 0.0337 | 0.0530 | 0.0613 | 0.0000 | 1.0000 | 1.0000 | 1.0000 | 1.0000 | 1.0000 | 1.0000 | 1.0000 | 1.0000 | 1.0000 | 1.0000 | 1.0000 | 1.0000 | 0.9705 | 0.9872 |  | 1.0000 | 0.9965 | 0.9072 | 0.9732 |
| **26** | 0.0000 | 0.0000 | 0.0000 | 0.0000 | 0.0000 | 0.0000 | 0.0000 | 0.0000 | 0.0000 | 0.0000 | 0.8722 | 1.0000 | 1.0000 | 1.0000 | 0.7650 | 0.9946 | 1.0000 | 1.0000 | 1.0000 | 0.9680 | 1.0000 | 1.0000 | 1.0000 | 1.0000 | 1.0000 |  | 1.0000 | 1.0000 | 1.0000 |
| **27** | 0.0000 | 0.0000 | 0.0000 | 0.0000 | 0.0000 | 0.0000 | 0.0000 | 0.0000 | 0.0000 | 0.0000 | 0.2403 | 0.9999 | 1.0000 | 1.0000 | 0.1389 | 0.7131 | 1.0000 | 1.0000 | 1.0000 | 0.4775 | 0.9998 | 1.0000 | 1.0000 | 1.0000 | 0.9965 | 1.0000 |  | 1.0000 | 1.0000 |
| **28** | 0.0000 | 0.0000 | 0.0000 | 0.0000 | 0.0000 | 0.0000 | 0.0000 | 0.0000 | 0.0000 | 0.0000 | 0.0310 | 0.9862 | 1.0000 | 0.9978 | 0.0128 | 0.2541 | 0.9932 | 1.0000 | 1.0000 | 0.1060 | 0.9810 | 1.0000 | 1.0000 | 1.0000 | 0.9072 | 1.0000 | 1.0000 |  | 1.0000 |
| **29** | 0.0000 | 0.0000 | 0.0000 | 0.0000 | 0.0000 | 0.0000 | 0.0000 | 0.0000 | 0.0000 | 0.0000 | 0.0888 | 0.9978 | 1.0000 | 0.9998 | 0.0428 | 0.4533 | 0.9991 | 1.0000 | 1.0000 | 0.2367 | 0.9967 | 1.0000 | 1.0000 | 1.0000 | 0.9732 | 1.0000 | 1.0000 | 1.0000 |  |

**Supplementary Table 2E***. post hoc* Scheffé test (p<0.05 marked in red) for daily sleep amount over time (days) during lights off.

| **Day** | **1** | **2** | **3** | **4** | **5** | **6** | **7** | **8** | **9** | **10** | **11** | **12** | **13** | **14** | **15** | **16** | **17** | **18** | **19** | **20** | **21** | **22** | **23** | **24** | **25** | **26** | **27** | **28** | **29** |
| --- | --- | --- | --- | --- | --- | --- | --- | --- | --- | --- | --- | --- | --- | --- | --- | --- | --- | --- | --- | --- | --- | --- | --- | --- | --- | --- | --- | --- | --- |
| **1** |  | 1.0000 | 0.9999 | 1.0000 | 0.9842 | 0.0023 | 0.0389 | 0.0028 | 0.2518 | 0.6371 | 0.0000 | 0.0000 | 0.0000 | 0.0000 | 0.0000 | 0.0000 | 0.0000 | 0.0000 | 0.0000 | 0.0000 | 0.0000 | 0.0000 | 0.0000 | 0.0000 | 0.0000 | 0.0000 | 0.0000 | 0.0000 | 0.0000 |
| **2** | 1.0000 |  | 1.0000 | 1.0000 | 1.0000 | 0.1450 | 0.5380 | 0.1622 | 0.9015 | 0.9924 | 0.0000 | 0.0000 | 0.0000 | 0.0000 | 0.0000 | 0.0000 | 0.0000 | 0.0000 | 0.0000 | 0.0000 | 0.0000 | 0.0000 | 0.0000 | 0.0000 | 0.0000 | 0.0000 | 0.0000 | 0.0000 | 0.0000 |
| **3** | 0.9999 | 1.0000 |  | 1.0000 | 1.0000 | 0.8444 | 0.9894 | 0.8628 | 0.9999 | 1.0000 | 0.0002 | 0.0000 | 0.0000 | 0.0000 | 0.0000 | 0.0000 | 0.0000 | 0.0000 | 0.0000 | 0.0000 | 0.0000 | 0.0000 | 0.0000 | 0.0000 | 0.0000 | 0.0000 | 0.0000 | 0.0000 | 0.0000 |
| **4** | 1.0000 | 1.0000 | 1.0000 |  | 1.0000 | 0.6964 | 0.9612 | 0.7231 | 0.9990 | 1.0000 | 0.0000 | 0.0000 | 0.0000 | 0.0000 | 0.0000 | 0.0000 | 0.0000 | 0.0000 | 0.0000 | 0.0000 | 0.0000 | 0.0000 | 0.0000 | 0.0000 | 0.0000 | 0.0000 | 0.0000 | 0.0000 | 0.0000 |
| **5** | 0.9842 | 1.0000 | 1.0000 | 1.0000 |  | 0.9937 | 1.0000 | 0.9951 | 1.0000 | 1.0000 | 0.0084 | 0.0000 | 0.0000 | 0.0000 | 0.0000 | 0.0000 | 0.0000 | 0.0000 | 0.0000 | 0.0000 | 0.0000 | 0.0000 | 0.0000 | 0.0000 | 0.0000 | 0.0000 | 0.0000 | 0.0000 | 0.0000 |
| **6** | 0.0023 | 0.1450 | 0.8444 | 0.6964 | 0.9937 |  | 1.0000 | 1.0000 | 1.0000 | 1.0000 | 0.9968 | 0.0012 | 0.0000 | 0.0009 | 0.0001 | 0.2492 | 0.0004 | 0.0693 | 0.0459 | 0.0009 | 0.4977 | 0.0048 | 0.0028 | 0.0052 | 0.0129 | 0.0748 | 0.0012 | 0.0000 | 0.0001 |
| **7** | 0.0389 | 0.5380 | 0.9894 | 0.9612 | 1.0000 | 1.0000 |  | 1.0000 | 1.0000 | 1.0000 | 0.9190 | 0.0000 | 0.0000 | 0.0000 | 0.0000 | 0.0363 | 0.0000 | 0.0051 | 0.0028 | 0.0000 | 0.1242 | 0.0001 | 0.0001 | 0.0002 | 0.0005 | 0.0057 | 0.0000 | 0.0000 | 0.0000 |
| **8** | 0.0028 | 0.1622 | 0.8628 | 0.7231 | 0.9951 | 1.0000 | 1.0000 |  | 1.0000 | 1.0000 | 0.9958 | 0.0010 | 0.0000 | 0.0007 | 0.0001 | 0.2265 | 0.0003 | 0.0604 | 0.0395 | 0.0007 | 0.4676 | 0.0039 | 0.0023 | 0.0043 | 0.0107 | 0.0652 | 0.0009 | 0.0000 | 0.0001 |
| **9** | 0.2518 | 0.9015 | 0.9999 | 0.9990 | 1.0000 | 1.0000 | 1.0000 | 1.0000 |  | 1.0000 | 0.5782 | 0.0000 | 0.0000 | 0.0000 | 0.0000 | 0.0022 | 0.0000 | 0.0002 | 0.0001 | 0.0000 | 0.0129 | 0.0000 | 0.0000 | 0.0000 | 0.0000 | 0.0002 | 0.0000 | 0.0000 | 0.0000 |
| **10** | 0.6371 | 0.9924 | 1.0000 | 1.0000 | 1.0000 | 1.0000 | 1.0000 | 1.0000 | 1.0000 |  | 0.2074 | 0.0000 | 0.0000 | 0.0000 | 0.0000 | 0.0001 | 0.0000 | 0.0000 | 0.0000 | 0.0000 | 0.0008 | 0.0000 | 0.0000 | 0.0000 | 0.0000 | 0.0000 | 0.0000 | 0.0000 | 0.0000 |
| **11** | 0.0000 | 0.0000 | 0.0002 | 0.0000 | 0.0084 | 0.9968 | 0.9190 | 0.9958 | 0.5782 | 0.2074 |  | 0.9546 | 0.5078 | 0.9451 | 0.7985 | 1.0000 | 0.9015 | 0.9999 | 0.9997 | 0.9420 | 1.0000 | 0.9876 | 0.9782 | 0.9888 | 0.9966 | 0.9999 | 0.9537 | 0.6112 | 0.7730 |
| **12** | 0.0000 | 0.0000 | 0.0000 | 0.0000 | 0.0000 | 0.0012 | 0.0000 | 0.0010 | 0.0000 | 0.0000 | 0.9546 |  | 1.0000 | 1.0000 | 1.0000 | 1.0000 | 1.0000 | 1.0000 | 1.0000 | 1.0000 | 1.0000 | 1.0000 | 1.0000 | 1.0000 | 1.0000 | 1.0000 | 1.0000 | 1.0000 | 1.0000 |
| **13** | 0.0000 | 0.0000 | 0.0000 | 0.0000 | 0.0000 | 0.0000 | 0.0000 | 0.0000 | 0.0000 | 0.0000 | 0.5078 | 1.0000 |  | 1.0000 | 1.0000 | 0.9998 | 1.0000 | 1.0000 | 1.0000 | 1.0000 | 0.9970 | 1.0000 | 1.0000 | 1.0000 | 1.0000 | 1.0000 | 1.0000 | 1.0000 | 1.0000 |
| **14** | 0.0000 | 0.0000 | 0.0000 | 0.0000 | 0.0000 | 0.0009 | 0.0000 | 0.0007 | 0.0000 | 0.0000 | 0.9451 | 1.0000 | 1.0000 |  | 1.0000 | 1.0000 | 1.0000 | 1.0000 | 1.0000 | 1.0000 | 1.0000 | 1.0000 | 1.0000 | 1.0000 | 1.0000 | 1.0000 | 1.0000 | 1.0000 | 1.0000 |
| **15** | 0.0000 | 0.0000 | 0.0000 | 0.0000 | 0.0000 | 0.0001 | 0.0000 | 0.0001 | 0.0000 | 0.0000 | 0.7985 | 1.0000 | 1.0000 | 1.0000 |  | 1.0000 | 1.0000 | 1.0000 | 1.0000 | 1.0000 | 0.9999 | 1.0000 | 1.0000 | 1.0000 | 1.0000 | 1.0000 | 1.0000 | 1.0000 | 1.0000 |
| **16** | 0.0000 | 0.0000 | 0.0000 | 0.0000 | 0.0000 | 0.2492 | 0.0363 | 0.2265 | 0.0022 | 0.0001 | 1.0000 | 1.0000 | 0.9998 | 1.0000 | 1.0000 |  | 1.0000 | 1.0000 | 1.0000 | 1.0000 | 1.0000 | 1.0000 | 1.0000 | 1.0000 | 1.0000 | 1.0000 | 1.0000 | 1.0000 | 1.0000 |
| **17** | 0.0000 | 0.0000 | 0.0000 | 0.0000 | 0.0000 | 0.0004 | 0.0000 | 0.0003 | 0.0000 | 0.0000 | 0.9015 | 1.0000 | 1.0000 | 1.0000 | 1.0000 | 1.0000 |  | 1.0000 | 1.0000 | 1.0000 | 1.0000 | 1.0000 | 1.0000 | 1.0000 | 1.0000 | 1.0000 | 1.0000 | 1.0000 | 1.0000 |
| **18** | 0.0000 | 0.0000 | 0.0000 | 0.0000 | 0.0000 | 0.0693 | 0.0051 | 0.0604 | 0.0002 | 0.0000 | 0.9999 | 1.0000 | 1.0000 | 1.0000 | 1.0000 | 1.0000 | 1.0000 |  | 1.0000 | 1.0000 | 1.0000 | 1.0000 | 1.0000 | 1.0000 | 1.0000 | 1.0000 | 1.0000 | 1.0000 | 1.0000 |
| **19** | 0.0000 | 0.0000 | 0.0000 | 0.0000 | 0.0000 | 0.0459 | 0.0028 | 0.0395 | 0.0001 | 0.0000 | 0.9997 | 1.0000 | 1.0000 | 1.0000 | 1.0000 | 1.0000 | 1.0000 | 1.0000 |  | 1.0000 | 1.0000 | 1.0000 | 1.0000 | 1.0000 | 1.0000 | 1.0000 | 1.0000 | 1.0000 | 1.0000 |
| **20** | 0.0000 | 0.0000 | 0.0000 | 0.0000 | 0.0000 | 0.0009 | 0.0000 | 0.0007 | 0.0000 | 0.0000 | 0.9420 | 1.0000 | 1.0000 | 1.0000 | 1.0000 | 1.0000 | 1.0000 | 1.0000 | 1.0000 |  | 1.0000 | 1.0000 | 1.0000 | 1.0000 | 1.0000 | 1.0000 | 1.0000 | 1.0000 | 1.0000 |
| **21** | 0.0000 | 0.0000 | 0.0000 | 0.0000 | 0.0000 | 0.4977 | 0.1242 | 0.4676 | 0.0129 | 0.0008 | 1.0000 | 1.0000 | 0.9970 | 1.0000 | 0.9999 | 1.0000 | 1.0000 | 1.0000 | 1.0000 | 1.0000 |  | 1.0000 | 1.0000 | 1.0000 | 1.0000 | 1.0000 | 1.0000 | 0.9989 | 0.9999 |
| **22** | 0.0000 | 0.0000 | 0.0000 | 0.0000 | 0.0000 | 0.0048 | 0.0001 | 0.0039 | 0.0000 | 0.0000 | 0.9876 | 1.0000 | 1.0000 | 1.0000 | 1.0000 | 1.0000 | 1.0000 | 1.0000 | 1.0000 | 1.0000 | 1.0000 |  | 1.0000 | 1.0000 | 1.0000 | 1.0000 | 1.0000 | 1.0000 | 1.0000 |
| **23** | 0.0000 | 0.0000 | 0.0000 | 0.0000 | 0.0000 | 0.0028 | 0.0001 | 0.0023 | 0.0000 | 0.0000 | 0.9782 | 1.0000 | 1.0000 | 1.0000 | 1.0000 | 1.0000 | 1.0000 | 1.0000 | 1.0000 | 1.0000 | 1.0000 | 1.0000 |  | 1.0000 | 1.0000 | 1.0000 | 1.0000 | 1.0000 | 1.0000 |
| **24** | 0.0000 | 0.0000 | 0.0000 | 0.0000 | 0.0000 | 0.0052 | 0.0002 | 0.0043 | 0.0000 | 0.0000 | 0.9888 | 1.0000 | 1.0000 | 1.0000 | 1.0000 | 1.0000 | 1.0000 | 1.0000 | 1.0000 | 1.0000 | 1.0000 | 1.0000 | 1.0000 |  | 1.0000 | 1.0000 | 1.0000 | 1.0000 | 1.0000 |
| **25** | 0.0000 | 0.0000 | 0.0000 | 0.0000 | 0.0000 | 0.0129 | 0.0005 | 0.0107 | 0.0000 | 0.0000 | 0.9966 | 1.0000 | 1.0000 | 1.0000 | 1.0000 | 1.0000 | 1.0000 | 1.0000 | 1.0000 | 1.0000 | 1.0000 | 1.0000 | 1.0000 | 1.0000 |  | 1.0000 | 1.0000 | 1.0000 | 1.0000 |
| **26** | 0.0000 | 0.0000 | 0.0000 | 0.0000 | 0.0000 | 0.0748 | 0.0057 | 0.0652 | 0.0002 | 0.0000 | 0.9999 | 1.0000 | 1.0000 | 1.0000 | 1.0000 | 1.0000 | 1.0000 | 1.0000 | 1.0000 | 1.0000 | 1.0000 | 1.0000 | 1.0000 | 1.0000 | 1.0000 |  | 1.0000 | 1.0000 | 1.0000 |
| **27** | 0.0000 | 0.0000 | 0.0000 | 0.0000 | 0.0000 | 0.0012 | 0.0000 | 0.0009 | 0.0000 | 0.0000 | 0.9537 | 1.0000 | 1.0000 | 1.0000 | 1.0000 | 1.0000 | 1.0000 | 1.0000 | 1.0000 | 1.0000 | 1.0000 | 1.0000 | 1.0000 | 1.0000 | 1.0000 | 1.0000 |  | 1.0000 | 1.0000 |
| **28** | 0.0000 | 0.0000 | 0.0000 | 0.0000 | 0.0000 | 0.0000 | 0.0000 | 0.0000 | 0.0000 | 0.0000 | 0.6112 | 1.0000 | 1.0000 | 1.0000 | 1.0000 | 1.0000 | 1.0000 | 1.0000 | 1.0000 | 1.0000 | 0.9989 | 1.0000 | 1.0000 | 1.0000 | 1.0000 | 1.0000 | 1.0000 |  | 1.0000 |
| **29** | 0.0000 | 0.0000 | 0.0000 | 0.0000 | 0.0000 | 0.0001 | 0.0000 | 0.0001 | 0.0000 | 0.0000 | 0.7730 | 1.0000 | 1.0000 | 1.0000 | 1.0000 | 1.0000 | 1.0000 | 1.0000 | 1.0000 | 1.0000 | 0.9999 | 1.0000 | 1.0000 | 1.0000 | 1.0000 | 1.0000 | 1.0000 | 1.0000 |  |

| **Day** | **1** | **2** | **3** | **4** | **5** | **6** | **7** | **8** | **9** | **10** | **11** | **12** | **13** | **14** | **15** | **16** | **17** | **18** | **19** | **20** | **21** | **22** | **23** | **24** | **25** | **26** | **27** | **28** | **29** |
| --- | --- | --- | --- | --- | --- | --- | --- | --- | --- | --- | --- | --- | --- | --- | --- | --- | --- | --- | --- | --- | --- | --- | --- | --- | --- | --- | --- | --- | --- |
| **1** |  | 1.0000 | 1.0000 | 1.0000 | 1.0000 | 1.0000 | 1.0000 | 1.0000 | 1.0000 | 1.0000 | 0.7917 | 0.2038 | 0.0112 | 0.0698 | 1.0000 | 0.8871 | 0.1866 | 0.0001 | 0.0003 | 1.0000 | 0.0044 | 0.0000 | 0.0000 | 0.0000 | 0.3618 | 0.0000 | 0.0000 | 0.0000 | 0.0000 |
| **2** | 1.0000 |  | 1.0000 | 1.0000 | 1.0000 | 1.0000 | 1.0000 | 1.0000 | 1.0000 | 1.0000 | 0.6872 | 0.1295 | 0.0051 | 0.0383 | 1.0000 | 0.8106 | 0.1169 | 0.0000 | 0.0001 | 0.9999 | 0.0019 | 0.0000 | 0.0000 | 0.0000 | 0.2545 | 0.0000 | 0.0000 | 0.0000 | 0.0000 |
| **3** | 1.0000 | 1.0000 |  | 1.0000 | 1.0000 | 0.9999 | 0.9984 | 0.9997 | 0.9486 | 1.0000 | 0.0792 | 0.0018 | 0.0000 | 0.0002 | 0.9999 | 0.1465 | 0.0015 | 0.0000 | 0.0000 | 0.9327 | 0.0000 | 0.0000 | 0.0000 | 0.0000 | 0.0066 | 0.0000 | 0.0000 | 0.0000 | 0.0000 |
| **4** | 1.0000 | 1.0000 | 1.0000 |  | 1.0000 | 1.0000 | 1.0000 | 1.0000 | 0.9976 | 1.0000 | 0.3421 | 0.0246 | 0.0004 | 0.0048 | 1.0000 | 0.4873 | 0.0214 | 0.0000 | 0.0000 | 0.9962 | 0.0001 | 0.0000 | 0.0000 | 0.0000 | 0.0652 | 0.0000 | 0.0000 | 0.0000 | 0.0000 |
| **5** | 1.0000 | 1.0000 | 1.0000 | 1.0000 |  | 0.9998 | 0.9973 | 0.9995 | 0.9319 | 1.0000 | 0.0626 | 0.0012 | 0.0000 | 0.0001 | 0.9998 | 0.1200 | 0.0010 | 0.0000 | 0.0000 | 0.9125 | 0.0000 | 0.0000 | 0.0000 | 0.0000 | 0.0047 | 0.0000 | 0.0000 | 0.0000 | 0.0000 |
| **6** | 1.0000 | 1.0000 | 0.9999 | 1.0000 | 0.9998 |  | 1.0000 | 1.0000 | 1.0000 | 0.9999 | 0.9970 | 0.8241 | 0.2630 | 0.5906 | 1.0000 | 0.9993 | 0.8059 | 0.0172 | 0.0300 | 1.0000 | 0.1613 | 0.0017 | 0.0000 | 0.0000 | 0.9264 | 0.0013 | 0.0000 | 0.0000 | 0.0000 |
| **7** | 1.0000 | 1.0000 | 0.9984 | 1.0000 | 0.9973 | 1.0000 |  | 1.0000 | 1.0000 | 0.9984 | 0.9998 | 0.9492 | 0.5067 | 0.8182 | 1.0000 | 1.0000 | 0.9414 | 0.0665 | 0.1034 | 1.0000 | 0.3638 | 0.0099 | 0.0000 | 0.0000 | 0.9854 | 0.0079 | 0.0002 | 0.0000 | 0.0001 |
| **8** | 1.0000 | 1.0000 | 0.9997 | 1.0000 | 0.9995 | 1.0000 | 1.0000 |  | 1.0000 | 0.9997 | 0.9989 | 0.8849 | 0.3499 | 0.6872 | 1.0000 | 0.9998 | 0.8709 | 0.0300 | 0.0501 | 1.0000 | 0.2283 | 0.0035 | 0.0000 | 0.0000 | 0.9579 | 0.0027 | 0.0000 | 0.0000 | 0.0000 |
| **9** | 1.0000 | 1.0000 | 0.9486 | 0.9976 | 0.9319 | 1.0000 | 1.0000 | 1.0000 |  | 0.9499 | 1.0000 | 0.9984 | 0.8849 | 0.9844 | 1.0000 | 1.0000 | 0.9980 | 0.3440 | 0.4402 | 1.0000 | 0.7917 | 0.1034 | 0.0002 | 0.0004 | 0.9998 | 0.0886 | 0.0055 | 0.0006 | 0.0022 |
| **10** | 1.0000 | 1.0000 | 1.0000 | 1.0000 | 1.0000 | 0.9999 | 0.9984 | 0.9997 | 0.9499 |  | 0.0807 | 0.0018 | 0.0000 | 0.0002 | 0.9999 | 0.1489 | 0.0015 | 0.0000 | 0.0000 | 0.9342 | 0.0000 | 0.0000 | 0.0000 | 0.0000 | 0.0068 | 0.0000 | 0.0000 | 0.0000 | 0.0000 |
| **11** | 0.7917 | 0.6872 | 0.0792 | 0.3421 | 0.0626 | 0.9970 | 0.9998 | 0.9989 | 1.0000 | 0.0807 |  | 1.0000 | 1.0000 | 1.0000 | 0.9978 | 1.0000 | 1.0000 | 0.9976 | 0.9991 | 1.0000 | 1.0000 | 0.9644 | 0.2159 | 0.2948 | 1.0000 | 0.9557 | 0.6327 | 0.3324 | 0.5024 |
| **12** | 0.2038 | 0.1295 | 0.0018 | 0.0246 | 0.0012 | 0.8241 | 0.9492 | 0.8849 | 0.9984 | 0.0018 | 1.0000 |  | 1.0000 | 1.0000 | 0.8466 | 1.0000 | 1.0000 | 1.0000 | 1.0000 | 0.9990 | 1.0000 | 0.9999 | 0.8044 | 0.8697 | 1.0000 | 0.9999 | 0.9816 | 0.8925 | 0.9573 |
| **13** | 0.0112 | 0.0051 | 0.0000 | 0.0004 | 0.0000 | 0.2630 | 0.5067 | 0.3499 | 0.8849 | 0.0000 | 1.0000 | 1.0000 |  | 1.0000 | 0.2912 | 1.0000 | 1.0000 | 1.0000 | 1.0000 | 0.9087 | 1.0000 | 1.0000 | 0.9948 | 0.9979 | 1.0000 | 1.0000 | 1.0000 | 0.9986 | 0.9998 |
| **14** | 0.0698 | 0.0383 | 0.0002 | 0.0048 | 0.0001 | 0.5906 | 0.8182 | 0.6872 | 0.9844 | 0.0002 | 1.0000 | 1.0000 | 1.0000 |  | 0.6243 | 1.0000 | 1.0000 | 1.0000 | 1.0000 | 0.9892 | 1.0000 | 1.0000 | 0.9435 | 0.9688 | 1.0000 | 1.0000 | 0.9980 | 0.9764 | 0.9936 |
| **15** | 1.0000 | 1.0000 | 0.9999 | 1.0000 | 0.9998 | 1.0000 | 1.0000 | 1.0000 | 1.0000 | 0.9999 | 0.9978 | 0.8466 | 0.2912 | 0.6243 |  | 0.9995 | 0.8299 | 0.0209 | 0.0359 | 1.0000 | 0.1824 | 0.0022 | 0.0000 | 0.0000 | 0.9386 | 0.0017 | 0.0000 | 0.0000 | 0.0000 |
| **16** | 0.8871 | 0.8106 | 0.1465 | 0.4873 | 0.1200 | 0.9993 | 1.0000 | 0.9998 | 1.0000 | 0.1489 | 1.0000 | 1.0000 | 1.0000 | 1.0000 | 0.9995 |  | 1.0000 | 0.9914 | 0.9963 | 1.0000 | 0.9999 | 0.9179 | 0.1252 | 0.1824 | 1.0000 | 0.9019 | 0.4830 | 0.2113 | 0.3558 |
| **17** | 0.1866 | 0.1169 | 0.0015 | 0.0214 | 0.0010 | 0.8059 | 0.9414 | 0.8709 | 0.9980 | 0.0015 | 1.0000 | 1.0000 | 1.0000 | 1.0000 | 0.8299 | 1.0000 |  | 1.0000 | 1.0000 | 0.9987 | 1.0000 | 0.9999 | 0.8226 | 0.8838 | 1.0000 | 0.9999 | 0.9847 | 0.9048 | 0.9634 |
| **18** | 0.0001 | 0.0000 | 0.0000 | 0.0000 | 0.0000 | 0.0172 | 0.0665 | 0.0300 | 0.3440 | 0.0000 | 0.9976 | 1.0000 | 1.0000 | 1.0000 | 0.0209 | 0.9914 | 1.0000 |  | 1.0000 | 0.3901 | 1.0000 | 1.0000 | 1.0000 | 1.0000 | 1.0000 | 1.0000 | 1.0000 | 1.0000 | 1.0000 |
| **19** | 0.0003 | 0.0001 | 0.0000 | 0.0000 | 0.0000 | 0.0300 | 0.1034 | 0.0501 | 0.4402 | 0.0000 | 0.9991 | 1.0000 | 1.0000 | 1.0000 | 0.0359 | 0.9963 | 1.0000 | 1.0000 |  | 0.4895 | 1.0000 | 1.0000 | 1.0000 | 1.0000 | 1.0000 | 1.0000 | 1.0000 | 1.0000 | 1.0000 |
| **20** | 1.0000 | 0.9999 | 0.9327 | 0.9962 | 0.9125 | 1.0000 | 1.0000 | 1.0000 | 1.0000 | 0.9342 | 1.0000 | 0.9990 | 0.9087 | 0.9892 | 1.0000 | 1.0000 | 0.9987 | 0.3901 | 0.4895 |  | 0.8270 | 0.1263 | 0.0003 | 0.0006 | 0.9999 | 0.1090 | 0.0075 | 0.0009 | 0.0032 |
| **21** | 0.0044 | 0.0019 | 0.0000 | 0.0001 | 0.0000 | 0.1613 | 0.3638 | 0.2283 | 0.7917 | 0.0000 | 1.0000 | 1.0000 | 1.0000 | 1.0000 | 0.1824 | 0.9999 | 1.0000 | 1.0000 | 1.0000 | 0.8270 |  | 1.0000 | 0.9987 | 0.9995 | 1.0000 | 1.0000 | 1.0000 | 0.9997 | 1.0000 |
| **22** | 0.0000 | 0.0000 | 0.0000 | 0.0000 | 0.0000 | 0.0017 | 0.0099 | 0.0035 | 0.1034 | 0.0000 | 0.9644 | 0.9999 | 1.0000 | 1.0000 | 0.0022 | 0.9179 | 0.9999 | 1.0000 | 1.0000 | 0.1263 | 1.0000 |  | 1.0000 | 1.0000 | 0.9991 | 1.0000 | 1.0000 | 1.0000 | 1.0000 |
| **23** | 0.0000 | 0.0000 | 0.0000 | 0.0000 | 0.0000 | 0.0000 | 0.0000 | 0.0000 | 0.0002 | 0.0000 | 0.2159 | 0.8044 | 0.9948 | 0.9435 | 0.0000 | 0.1252 | 0.8226 | 1.0000 | 1.0000 | 0.0003 | 0.9987 | 1.0000 |  | 1.0000 | 0.6389 | 1.0000 | 1.0000 | 1.0000 | 1.0000 |
| **24** | 0.0000 | 0.0000 | 0.0000 | 0.0000 | 0.0000 | 0.0000 | 0.0000 | 0.0000 | 0.0004 | 0.0000 | 0.2948 | 0.8697 | 0.9979 | 0.9688 | 0.0000 | 0.1824 | 0.8838 | 1.0000 | 1.0000 | 0.0006 | 0.9995 | 1.0000 | 1.0000 |  | 0.7310 | 1.0000 | 1.0000 | 1.0000 | 1.0000 |
| **25** | 0.3618 | 0.2545 | 0.0066 | 0.0652 | 0.0047 | 0.9264 | 0.9854 | 0.9579 | 0.9998 | 0.0068 | 1.0000 | 1.0000 | 1.0000 | 1.0000 | 0.9386 | 1.0000 | 1.0000 | 1.0000 | 1.0000 | 0.9999 | 1.0000 | 0.9991 | 0.6389 | 0.7310 |  | 0.9988 | 0.9393 | 0.7666 | 0.8838 |
| **26** | 0.0000 | 0.0000 | 0.0000 | 0.0000 | 0.0000 | 0.0013 | 0.0079 | 0.0027 | 0.0886 | 0.0000 | 0.9557 | 0.9999 | 1.0000 | 1.0000 | 0.0017 | 0.9019 | 0.9999 | 1.0000 | 1.0000 | 0.1090 | 1.0000 | 1.0000 | 1.0000 | 1.0000 | 0.9988 |  | 1.0000 | 1.0000 | 1.0000 |
| **27** | 0.0000 | 0.0000 | 0.0000 | 0.0000 | 0.0000 | 0.0000 | 0.0002 | 0.0000 | 0.0055 | 0.0000 | 0.6327 | 0.9816 | 1.0000 | 0.9980 | 0.0000 | 0.4830 | 0.9847 | 1.0000 | 1.0000 | 0.0075 | 1.0000 | 1.0000 | 1.0000 | 1.0000 | 0.9393 | 1.0000 |  | 1.0000 | 1.0000 |
| **28** | 0.0000 | 0.0000 | 0.0000 | 0.0000 | 0.0000 | 0.0000 | 0.0000 | 0.0000 | 0.0006 | 0.0000 | 0.3324 | 0.8925 | 0.9986 | 0.9764 | 0.0000 | 0.2113 | 0.9048 | 1.0000 | 1.0000 | 0.0009 | 0.9997 | 1.0000 | 1.0000 | 1.0000 | 0.7666 | 1.0000 | 1.0000 |  | 1.0000 |
| **29** | 0.0000 | 0.0000 | 0.0000 | 0.0000 | 0.0000 | 0.0000 | 0.0001 | 0.0000 | 0.0022 | 0.0000 | 0.5024 | 0.9573 | 0.9998 | 0.9936 | 0.0000 | 0.3558 | 0.9634 | 1.0000 | 1.0000 | 0.0032 | 1.0000 | 1.0000 | 1.0000 | 1.0000 | 0.8838 | 1.0000 | 1.0000 | 1.0000 |  |

**Supplementary Table 2F***. post hoc* Scheffé test (p<0.05 marked in red) for daily sleep amount over time (days) during lights on.
