# Supplementary material for: Sleep timing in flies from “adolescence” to adulthood
Source: Fly (Austin). 2024 Dec 30;19(1):2448022. doi: 10.1080/19336934.2024.2448022 (PMC11702927; doi:10.1080/19336934.2024.2448022)
Supplement: FliesAge_Suppl_Table4.docx [file KFLY_A_2448022_SM2379.docx]

**Supplementary Table 4A***. post hoc* Scheffé test (p<0.05 marked in red) for sleep onset over time (days).

| **Day** | **1** | **2** | **3** | **4** | **5** | **6** | **7** | **8** | **9** | **10** | **11** | **12** | **13** | **14** | **15** | **16** | **17** | **18** | **19** | **20** | **21** | **22** | **23** | **24** | **25** | **26** | **27** | **28** | **29** | **30** |
| --- | --- | --- | --- | --- | --- | --- | --- | --- | --- | --- | --- | --- | --- | --- | --- | --- | --- | --- | --- | --- | --- | --- | --- | --- | --- | --- | --- | --- | --- | --- |
| **1** |  | 1.0000 | 0.9959 | 0.8041 | 0.9992 | 0.9999 | 0.9998 | 1.0000 | 1.0000 | 1.0000 | 1.0000 | 1.0000 | 1.0000 | 1.0000 | 1.0000 | 1.0000 | 1.0000 | 1.0000 | 0.9999 | 0.9395 | 1.0000 | 0.9639 | 0.9590 | 0.8299 | 0.8041 | 0.9713 | 0.6262 | 0.3269 | 0.1579 | 0.2161 |
| **2** | 1.0000 |  | 1.0000 | 1.0000 | 1.0000 | 1.0000 | 1.0000 | 1.0000 | 1.0000 | 1.0000 | 1.0000 | 0.9946 | 0.9590 | 0.7192 | 0.6004 | 0.9759 | 0.9359 | 0.8536 | 0.5742 | 0.0450 | 0.9202 | 0.0690 | 0.0626 | 0.0134 | 0.0108 | 0.0814 | 0.0028 | 0.0003 | 0.0000 | 0.0001 |
| **3** | 0.9959 | 1.0000 |  | 1.0000 | 1.0000 | 1.0000 | 1.0000 | 1.0000 | 1.0000 | 1.0000 | 1.0000 | 0.7238 | 0.4471 | 0.1163 | 0.0673 | 0.5318 | 0.3704 | 0.2237 | 0.0595 | 0.0004 | 0.3316 | 0.0009 | 0.0007 | 0.0001 | 0.0000 | 0.0012 | 0.0000 | 0.0000 | 0.0000 | 0.0000 |
| **4** | 0.8041 | 1.0000 | 1.0000 |  | 1.0000 | 1.0000 | 1.0000 | 0.9994 | 0.9759 | 1.0000 | 0.9953 | 0.1548 | 0.0450 | 0.0035 | 0.0014 | 0.0673 | 0.0300 | 0.0111 | 0.0012 | 0.0000 | 0.0238 | 0.0000 | 0.0000 | 0.0000 | 0.0000 | 0.0000 | 0.0000 | 0.0000 | 0.0000 | 0.0000 |
| **5** | 0.9992 | 1.0000 | 1.0000 | 1.0000 |  | 1.0000 | 1.0000 | 1.0000 | 1.0000 | 1.0000 | 1.0000 | 0.8536 | 0.6211 | 0.2199 | 0.1402 | 0.7004 | 0.5424 | 0.3704 | 0.1265 | 0.0017 | 0.4999 | 0.0031 | 0.0027 | 0.0003 | 0.0002 | 0.0040 | 0.0000 | 0.0000 | 0.0000 | 0.0000 |
| **6** | 0.9999 | 1.0000 | 1.0000 | 1.0000 | 1.0000 |  | 1.0000 | 1.0000 | 1.0000 | 1.0000 | 1.0000 | 0.9322 | 0.7678 | 0.3557 | 0.2475 | 0.8299 | 0.7004 | 0.5318 | 0.2276 | 0.0052 | 0.6615 | 0.0092 | 0.0080 | 0.0011 | 0.0008 | 0.0115 | 0.0002 | 0.0000 | 0.0000 | 0.0000 |
| **7** | 0.9998 | 1.0000 | 1.0000 | 1.0000 | 1.0000 | 1.0000 |  | 1.0000 | 1.0000 | 1.0000 | 1.0000 | 0.9202 | 0.7418 | 0.3269 | 0.2237 | 0.8079 | 0.6714 | 0.4999 | 0.2050 | 0.0042 | 0.6313 | 0.0075 | 0.0066 | 0.0008 | 0.0006 | 0.0095 | 0.0001 | 0.0000 | 0.0000 | 0.0000 |
| **8** | 1.0000 | 1.0000 | 1.0000 | 0.9994 | 1.0000 | 1.0000 | 1.0000 |  | 1.0000 | 1.0000 | 1.0000 | 1.0000 | 0.9988 | 0.9627 | 0.9244 | 0.9995 | 0.9974 | 0.9890 | 0.9136 | 0.2684 | 0.9963 | 0.3460 | 0.3269 | 0.1239 | 0.1067 | 0.3804 | 0.0416 | 0.0073 | 0.0015 | 0.0029 |
| **9** | 1.0000 | 1.0000 | 1.0000 | 0.9759 | 1.0000 | 1.0000 | 1.0000 | 1.0000 |  | 1.0000 | 1.0000 | 1.0000 | 1.0000 | 0.9987 | 0.9959 | 1.0000 | 1.0000 | 0.9998 | 0.9948 | 0.6763 | 1.0000 | 0.7550 | 0.7374 | 0.4576 | 0.4211 | 0.7843 | 0.2395 | 0.0741 | 0.0238 | 0.0384 |
| **10** | 1.0000 | 1.0000 | 1.0000 | 1.0000 | 1.0000 | 1.0000 | 1.0000 | 1.0000 | 1.0000 |  | 1.0000 | 0.9844 | 0.9136 | 0.5899 | 0.4628 | 0.9445 | 0.8750 | 0.7550 | 0.4367 | 0.0218 | 0.8503 | 0.0354 | 0.0317 | 0.0057 | 0.0045 | 0.0427 | 0.0010 | 0.0001 | 0.0000 | 0.0000 |
| **11** | 1.0000 | 1.0000 | 1.0000 | 0.9953 | 1.0000 | 1.0000 | 1.0000 | 1.0000 | 1.0000 | 1.0000 |  | 1.0000 | 0.9999 | 0.9914 | 0.9783 | 1.0000 | 0.9997 | 0.9982 | 0.9741 | 0.4628 | 0.9995 | 0.5530 | 0.5318 | 0.2600 | 0.2315 | 0.5899 | 0.1090 | 0.0253 | 0.0066 | 0.0115 |
| **12** | 1.0000 | 0.9946 | 0.7238 | 0.1548 | 0.8536 | 0.9322 | 0.9202 | 1.0000 | 1.0000 | 0.9844 | 1.0000 |  | 1.0000 | 1.0000 | 1.0000 | 1.0000 | 1.0000 | 1.0000 | 1.0000 | 0.9999 | 1.0000 | 1.0000 | 1.0000 | 0.9989 | 0.9984 | 1.0000 | 0.9902 | 0.9303 | 0.8079 | 0.8661 |
| **13** | 1.0000 | 0.9590 | 0.4471 | 0.0450 | 0.6211 | 0.7678 | 0.7418 | 0.9988 | 1.0000 | 0.9136 | 0.9999 | 1.0000 |  | 1.0000 | 1.0000 | 1.0000 | 1.0000 | 1.0000 | 1.0000 | 1.0000 | 1.0000 | 1.0000 | 1.0000 | 1.0000 | 0.9999 | 1.0000 | 0.9993 | 0.9885 | 0.9492 | 0.9704 |
| **14** | 1.0000 | 0.7192 | 0.1163 | 0.0035 | 0.2199 | 0.3557 | 0.3269 | 0.9627 | 0.9987 | 0.5899 | 0.9914 | 1.0000 | 1.0000 |  | 1.0000 | 1.0000 | 1.0000 | 1.0000 | 1.0000 | 1.0000 | 1.0000 | 1.0000 | 1.0000 | 1.0000 | 1.0000 | 1.0000 | 1.0000 | 0.9998 | 0.9980 | 0.9992 |
| **15** | 1.0000 | 0.6004 | 0.0673 | 0.0014 | 0.1402 | 0.2475 | 0.2237 | 0.9244 | 0.9959 | 0.4628 | 0.9783 | 1.0000 | 1.0000 | 1.0000 |  | 1.0000 | 1.0000 | 1.0000 | 1.0000 | 1.0000 | 1.0000 | 1.0000 | 1.0000 | 1.0000 | 1.0000 | 1.0000 | 1.0000 | 1.0000 | 0.9994 | 0.9998 |
| **16** | 1.0000 | 0.9759 | 0.5318 | 0.0673 | 0.7004 | 0.8299 | 0.8079 | 0.9995 | 1.0000 | 0.9445 | 1.0000 | 1.0000 | 1.0000 | 1.0000 | 1.0000 |  | 1.0000 | 1.0000 | 1.0000 | 1.0000 | 1.0000 | 1.0000 | 1.0000 | 0.9999 | 0.9998 | 1.0000 | 0.9983 | 0.9791 | 0.9202 | 0.9507 |
| **17** | 1.0000 | 0.9359 | 0.3704 | 0.0300 | 0.5424 | 0.7004 | 0.6714 | 0.9974 | 1.0000 | 0.8750 | 0.9997 | 1.0000 | 1.0000 | 1.0000 | 1.0000 | 1.0000 |  | 1.0000 | 1.0000 | 1.0000 | 1.0000 | 1.0000 | 1.0000 | 1.0000 | 1.0000 | 1.0000 | 0.9997 | 0.9939 | 0.9683 | 0.9825 |
| **18** | 1.0000 | 0.8536 | 0.2237 | 0.0111 | 0.3704 | 0.5318 | 0.4999 | 0.9890 | 0.9998 | 0.7550 | 0.9982 | 1.0000 | 1.0000 | 1.0000 | 1.0000 | 1.0000 | 1.0000 |  | 1.0000 | 1.0000 | 1.0000 | 1.0000 | 1.0000 | 1.0000 | 1.0000 | 1.0000 | 1.0000 | 0.9987 | 0.9906 | 0.9955 |
| **19** | 0.9999 | 0.5742 | 0.0595 | 0.0012 | 0.1265 | 0.2276 | 0.2050 | 0.9136 | 0.9948 | 0.4367 | 0.9741 | 1.0000 | 1.0000 | 1.0000 | 1.0000 | 1.0000 | 1.0000 | 1.0000 |  | 1.0000 | 1.0000 | 1.0000 | 1.0000 | 1.0000 | 1.0000 | 1.0000 | 1.0000 | 1.0000 | 0.9996 | 0.9999 |
| **20** | 0.9395 | 0.0450 | 0.0004 | 0.0000 | 0.0017 | 0.0052 | 0.0042 | 0.2684 | 0.6763 | 0.0218 | 0.4628 | 0.9999 | 1.0000 | 1.0000 | 1.0000 | 1.0000 | 1.0000 | 1.0000 | 1.0000 |  | 1.0000 | 1.0000 | 1.0000 | 1.0000 | 1.0000 | 1.0000 | 1.0000 | 1.0000 | 1.0000 | 1.0000 |
| **21** | 1.0000 | 0.9202 | 0.3316 | 0.0238 | 0.4999 | 0.6615 | 0.6313 | 0.9963 | 1.0000 | 0.8503 | 0.9995 | 1.0000 | 1.0000 | 1.0000 | 1.0000 | 1.0000 | 1.0000 | 1.0000 | 1.0000 | 1.0000 |  | 1.0000 | 1.0000 | 1.0000 | 1.0000 | 1.0000 | 0.9998 | 0.9957 | 0.9759 | 0.9871 |
| **22** | 0.9639 | 0.0690 | 0.0009 | 0.0000 | 0.0031 | 0.0092 | 0.0075 | 0.3460 | 0.7550 | 0.0354 | 0.5530 | 1.0000 | 1.0000 | 1.0000 | 1.0000 | 1.0000 | 1.0000 | 1.0000 | 1.0000 | 1.0000 | 1.0000 |  | 1.0000 | 1.0000 | 1.0000 | 1.0000 | 1.0000 | 1.0000 | 1.0000 | 1.0000 |
| **23** | 0.9590 | 0.0626 | 0.0007 | 0.0000 | 0.0027 | 0.0080 | 0.0066 | 0.3269 | 0.7374 | 0.0317 | 0.5318 | 1.0000 | 1.0000 | 1.0000 | 1.0000 | 1.0000 | 1.0000 | 1.0000 | 1.0000 | 1.0000 | 1.0000 | 1.0000 |  | 1.0000 | 1.0000 | 1.0000 | 1.0000 | 1.0000 | 1.0000 | 1.0000 |
| **24** | 0.8299 | 0.0134 | 0.0001 | 0.0000 | 0.0003 | 0.0011 | 0.0008 | 0.1239 | 0.4576 | 0.0057 | 0.2600 | 0.9989 | 1.0000 | 1.0000 | 1.0000 | 0.9999 | 1.0000 | 1.0000 | 1.0000 | 1.0000 | 1.0000 | 1.0000 | 1.0000 |  | 1.0000 | 1.0000 | 1.0000 | 1.0000 | 1.0000 | 1.0000 |
| **25** | 0.8041 | 0.0108 | 0.0000 | 0.0000 | 0.0002 | 0.0008 | 0.0006 | 0.1067 | 0.4211 | 0.0045 | 0.2315 | 0.9984 | 0.9999 | 1.0000 | 1.0000 | 0.9998 | 1.0000 | 1.0000 | 1.0000 | 1.0000 | 1.0000 | 1.0000 | 1.0000 | 1.0000 |  | 1.0000 | 1.0000 | 1.0000 | 1.0000 | 1.0000 |
| **26** | 0.9713 | 0.0814 | 0.0012 | 0.0000 | 0.0040 | 0.0115 | 0.0095 | 0.3804 | 0.7843 | 0.0427 | 0.5899 | 1.0000 | 1.0000 | 1.0000 | 1.0000 | 1.0000 | 1.0000 | 1.0000 | 1.0000 | 1.0000 | 1.0000 | 1.0000 | 1.0000 | 1.0000 | 1.0000 |  | 1.0000 | 1.0000 | 1.0000 | 1.0000 |
| **27** | 0.6262 | 0.0028 | 0.0000 | 0.0000 | 0.0000 | 0.0002 | 0.0001 | 0.0416 | 0.2395 | 0.0010 | 0.1090 | 0.9902 | 0.9993 | 1.0000 | 1.0000 | 0.9983 | 0.9997 | 1.0000 | 1.0000 | 1.0000 | 0.9998 | 1.0000 | 1.0000 | 1.0000 | 1.0000 | 1.0000 |  | 1.0000 | 1.0000 | 1.0000 |
| **28** | 0.3269 | 0.0003 | 0.0000 | 0.0000 | 0.0000 | 0.0000 | 0.0000 | 0.0073 | 0.0741 | 0.0001 | 0.0253 | 0.9303 | 0.9885 | 0.9998 | 1.0000 | 0.9791 | 0.9939 | 0.9987 | 1.0000 | 1.0000 | 0.9957 | 1.0000 | 1.0000 | 1.0000 | 1.0000 | 1.0000 | 1.0000 |  | 1.0000 | 1.0000 |
| **29** | 0.1579 | 0.0000 | 0.0000 | 0.0000 | 0.0000 | 0.0000 | 0.0000 | 0.0015 | 0.0238 | 0.0000 | 0.0066 | 0.8079 | 0.9492 | 0.9980 | 0.9994 | 0.9202 | 0.9683 | 0.9906 | 0.9996 | 1.0000 | 0.9759 | 1.0000 | 1.0000 | 1.0000 | 1.0000 | 1.0000 | 1.0000 | 1.0000 |  | 1.0000 |
| **30** | 0.2161 | 0.0001 | 0.0000 | 0.0000 | 0.0000 | 0.0000 | 0.0000 | 0.0029 | 0.0384 | 0.0000 | 0.0115 | 0.8661 | 0.9704 | 0.9992 | 0.9998 | 0.9507 | 0.9825 | 0.9955 | 0.9999 | 1.0000 | 0.9871 | 1.0000 | 1.0000 | 1.0000 | 1.0000 | 1.0000 | 1.0000 | 1.0000 | 1.0000 |  |

**Supplementary Table 4B***. post hoc* Scheffé test (p<0.05 marked in red) for sleep offset over time (days).

| **Day** | **1** | **2** | **3** | **4** | **5** | **6** | **7** | **8** | **9** | **10** | **11** | **12** | **13** | **14** | **15** | **16** | **17** | **18** | **19** | **20** | **21** | **22** | **23** | **24** | **25** | **26** | **27** | **28** | **29** | **30** |
| --- | --- | --- | --- | --- | --- | --- | --- | --- | --- | --- | --- | --- | --- | --- | --- | --- | --- | --- | --- | --- | --- | --- | --- | --- | --- | --- | --- | --- | --- | --- |
| **1** |  | 0.8023 | 0.0001 | 0.1121 | 0.0066 | 0.0000 | 0.0001 | 0.0000 | 0.0000 | 0.0000 | 0.0000 | 0.0000 | 0.0000 | 0.0000 | 0.0000 | 0.0000 | 0.0000 | 0.0000 | 0.0000 | 0.0000 | 0.0000 | 0.0000 | 0.0000 | 0.0000 | 0.0000 | 0.0000 | 0.0000 | 0.0000 | 0.0000 | 0.0000 |
| **2** | 0.8023 |  | 0.9982 | 1.0000 | 1.0000 | 0.2325 | 0.9968 | 0.8620 | 0.9909 | 0.9858 | 0.4029 | 0.1233 | 0.0048 | 0.0344 | 0.0614 | 0.0053 | 0.0024 | 0.1233 | 0.1524 | 0.5830 | 0.3402 | 0.5869 | 0.7469 | 0.1501 | 0.2070 | 0.2070 | 0.1686 | 0.0045 | 0.2535 | 0.0519 |
| **3** | 0.0001 | 0.9982 |  | 1.0000 | 1.0000 | 1.0000 | 1.0000 | 1.0000 | 1.0000 | 1.0000 | 1.0000 | 1.0000 | 0.9862 | 0.9993 | 0.9998 | 0.9877 | 0.9729 | 1.0000 | 1.0000 | 1.0000 | 1.0000 | 1.0000 | 1.0000 | 1.0000 | 1.0000 | 1.0000 | 1.0000 | 0.9854 | 1.0000 | 0.9997 |
| **4** | 0.1121 | 1.0000 | 1.0000 |  | 1.0000 | 0.9169 | 1.0000 | 0.9997 | 1.0000 | 1.0000 | 0.9729 | 0.8191 | 0.2598 | 0.5751 | 0.6885 | 0.2724 | 0.1884 | 0.8191 | 0.8549 | 0.9927 | 0.9591 | 0.9929 | 0.9985 | 0.8525 | 0.9013 | 0.9013 | 0.8710 | 0.2535 | 0.9278 | 0.6556 |
| **5** | 0.0066 | 1.0000 | 1.0000 | 1.0000 |  | 0.9982 | 1.0000 | 1.0000 | 1.0000 | 1.0000 | 0.9998 | 0.9917 | 0.7630 | 0.9463 | 0.9736 | 0.7755 | 0.6777 | 0.9917 | 0.9947 | 1.0000 | 0.9995 | 1.0000 | 1.0000 | 0.9945 | 0.9975 | 0.9975 | 0.9958 | 0.7566 | 0.9986 | 0.9670 |
| **6** | 0.0000 | 0.2325 | 1.0000 | 0.9169 | 0.9982 |  | 1.0000 | 1.0000 | 1.0000 | 1.0000 | 1.0000 | 1.0000 | 1.0000 | 1.0000 | 1.0000 | 1.0000 | 1.0000 | 1.0000 | 1.0000 | 1.0000 | 1.0000 | 1.0000 | 1.0000 | 1.0000 | 1.0000 | 1.0000 | 1.0000 | 1.0000 | 1.0000 | 1.0000 |
| **7** | 0.0001 | 0.9968 | 1.0000 | 1.0000 | 1.0000 | 1.0000 |  | 1.0000 | 1.0000 | 1.0000 | 1.0000 | 1.0000 | 0.9914 | 0.9996 | 0.9999 | 0.9924 | 0.9823 | 1.0000 | 1.0000 | 1.0000 | 1.0000 | 1.0000 | 1.0000 | 1.0000 | 1.0000 | 1.0000 | 1.0000 | 0.9909 | 1.0000 | 0.9999 |
| **8** | 0.0000 | 0.8620 | 1.0000 | 0.9997 | 1.0000 | 1.0000 | 1.0000 |  | 1.0000 | 1.0000 | 1.0000 | 1.0000 | 1.0000 | 1.0000 | 1.0000 | 1.0000 | 0.9999 | 1.0000 | 1.0000 | 1.0000 | 1.0000 | 1.0000 | 1.0000 | 1.0000 | 1.0000 | 1.0000 | 1.0000 | 1.0000 | 1.0000 | 1.0000 |
| **9** | 0.0000 | 0.9909 | 1.0000 | 1.0000 | 1.0000 | 1.0000 | 1.0000 | 1.0000 |  | 1.0000 | 1.0000 | 1.0000 | 0.9970 | 0.9999 | 1.0000 | 0.9974 | 0.9931 | 1.0000 | 1.0000 | 1.0000 | 1.0000 | 1.0000 | 1.0000 | 1.0000 | 1.0000 | 1.0000 | 1.0000 | 0.9968 | 1.0000 | 1.0000 |
| **10** | 0.0000 | 0.9858 | 1.0000 | 1.0000 | 1.0000 | 1.0000 | 1.0000 | 1.0000 | 1.0000 |  | 1.0000 | 1.0000 | 0.9983 | 1.0000 | 1.0000 | 0.9985 | 0.9958 | 1.0000 | 1.0000 | 1.0000 | 1.0000 | 1.0000 | 1.0000 | 1.0000 | 1.0000 | 1.0000 | 1.0000 | 0.9982 | 1.0000 | 1.0000 |
| **11** | 0.0000 | 0.4029 | 1.0000 | 0.9729 | 0.9998 | 1.0000 | 1.0000 | 1.0000 | 1.0000 | 1.0000 |  | 1.0000 | 1.0000 | 1.0000 | 1.0000 | 1.0000 | 1.0000 | 1.0000 | 1.0000 | 1.0000 | 1.0000 | 1.0000 | 1.0000 | 1.0000 | 1.0000 | 1.0000 | 1.0000 | 1.0000 | 1.0000 | 1.0000 |
| **12** | 0.0000 | 0.1233 | 1.0000 | 0.8191 | 0.9917 | 1.0000 | 1.0000 | 1.0000 | 1.0000 | 1.0000 | 1.0000 |  | 1.0000 | 1.0000 | 1.0000 | 1.0000 | 1.0000 | 1.0000 | 1.0000 | 1.0000 | 1.0000 | 1.0000 | 1.0000 | 1.0000 | 1.0000 | 1.0000 | 1.0000 | 1.0000 | 1.0000 | 1.0000 |
| **13** | 0.0000 | 0.0048 | 0.9862 | 0.2598 | 0.7630 | 1.0000 | 0.9914 | 1.0000 | 0.9970 | 0.9983 | 1.0000 | 1.0000 |  | 1.0000 | 1.0000 | 1.0000 | 1.0000 | 1.0000 | 1.0000 | 1.0000 | 1.0000 | 1.0000 | 1.0000 | 1.0000 | 1.0000 | 1.0000 | 1.0000 | 1.0000 | 1.0000 | 1.0000 |
| **14** | 0.0000 | 0.0344 | 0.9993 | 0.5751 | 0.9463 | 1.0000 | 0.9996 | 1.0000 | 0.9999 | 1.0000 | 1.0000 | 1.0000 | 1.0000 |  | 1.0000 | 1.0000 | 1.0000 | 1.0000 | 1.0000 | 1.0000 | 1.0000 | 1.0000 | 1.0000 | 1.0000 | 1.0000 | 1.0000 | 1.0000 | 1.0000 | 1.0000 | 1.0000 |
| **15** | 0.0000 | 0.0614 | 0.9998 | 0.6885 | 0.9736 | 1.0000 | 0.9999 | 1.0000 | 1.0000 | 1.0000 | 1.0000 | 1.0000 | 1.0000 | 1.0000 |  | 1.0000 | 1.0000 | 1.0000 | 1.0000 | 1.0000 | 1.0000 | 1.0000 | 1.0000 | 1.0000 | 1.0000 | 1.0000 | 1.0000 | 1.0000 | 1.0000 | 1.0000 |
| **16** | 0.0000 | 0.0053 | 0.9877 | 0.2724 | 0.7755 | 1.0000 | 0.9924 | 1.0000 | 0.9974 | 0.9985 | 1.0000 | 1.0000 | 1.0000 | 1.0000 | 1.0000 |  | 1.0000 | 1.0000 | 1.0000 | 1.0000 | 1.0000 | 1.0000 | 1.0000 | 1.0000 | 1.0000 | 1.0000 | 1.0000 | 1.0000 | 1.0000 | 1.0000 |
| **17** | 0.0000 | 0.0024 | 0.9729 | 0.1884 | 0.6777 | 1.0000 | 0.9823 | 0.9999 | 0.9931 | 0.9958 | 1.0000 | 1.0000 | 1.0000 | 1.0000 | 1.0000 | 1.0000 |  | 1.0000 | 1.0000 | 1.0000 | 1.0000 | 1.0000 | 1.0000 | 1.0000 | 1.0000 | 1.0000 | 1.0000 | 1.0000 | 1.0000 | 1.0000 |
| **18** | 0.0000 | 0.1233 | 1.0000 | 0.8191 | 0.9917 | 1.0000 | 1.0000 | 1.0000 | 1.0000 | 1.0000 | 1.0000 | 1.0000 | 1.0000 | 1.0000 | 1.0000 | 1.0000 | 1.0000 |  | 1.0000 | 1.0000 | 1.0000 | 1.0000 | 1.0000 | 1.0000 | 1.0000 | 1.0000 | 1.0000 | 1.0000 | 1.0000 | 1.0000 |
| **19** | 0.0000 | 0.1524 | 1.0000 | 0.8549 | 0.9947 | 1.0000 | 1.0000 | 1.0000 | 1.0000 | 1.0000 | 1.0000 | 1.0000 | 1.0000 | 1.0000 | 1.0000 | 1.0000 | 1.0000 | 1.0000 |  | 1.0000 | 1.0000 | 1.0000 | 1.0000 | 1.0000 | 1.0000 | 1.0000 | 1.0000 | 1.0000 | 1.0000 | 1.0000 |
| **20** | 0.0000 | 0.5830 | 1.0000 | 0.9927 | 1.0000 | 1.0000 | 1.0000 | 1.0000 | 1.0000 | 1.0000 | 1.0000 | 1.0000 | 1.0000 | 1.0000 | 1.0000 | 1.0000 | 1.0000 | 1.0000 | 1.0000 |  | 1.0000 | 1.0000 | 1.0000 | 1.0000 | 1.0000 | 1.0000 | 1.0000 | 1.0000 | 1.0000 | 1.0000 |
| **21** | 0.0000 | 0.3402 | 1.0000 | 0.9591 | 0.9995 | 1.0000 | 1.0000 | 1.0000 | 1.0000 | 1.0000 | 1.0000 | 1.0000 | 1.0000 | 1.0000 | 1.0000 | 1.0000 | 1.0000 | 1.0000 | 1.0000 | 1.0000 |  | 1.0000 | 1.0000 | 1.0000 | 1.0000 | 1.0000 | 1.0000 | 1.0000 | 1.0000 | 1.0000 |
| **22** | 0.0000 | 0.5869 | 1.0000 | 0.9929 | 1.0000 | 1.0000 | 1.0000 | 1.0000 | 1.0000 | 1.0000 | 1.0000 | 1.0000 | 1.0000 | 1.0000 | 1.0000 | 1.0000 | 1.0000 | 1.0000 | 1.0000 | 1.0000 | 1.0000 |  | 1.0000 | 1.0000 | 1.0000 | 1.0000 | 1.0000 | 1.0000 | 1.0000 | 1.0000 |
| **23** | 0.0000 | 0.7469 | 1.0000 | 0.9985 | 1.0000 | 1.0000 | 1.0000 | 1.0000 | 1.0000 | 1.0000 | 1.0000 | 1.0000 | 1.0000 | 1.0000 | 1.0000 | 1.0000 | 1.0000 | 1.0000 | 1.0000 | 1.0000 | 1.0000 | 1.0000 |  | 1.0000 | 1.0000 | 1.0000 | 1.0000 | 1.0000 | 1.0000 | 1.0000 |
| **24** | 0.0000 | 0.1501 | 1.0000 | 0.8525 | 0.9945 | 1.0000 | 1.0000 | 1.0000 | 1.0000 | 1.0000 | 1.0000 | 1.0000 | 1.0000 | 1.0000 | 1.0000 | 1.0000 | 1.0000 | 1.0000 | 1.0000 | 1.0000 | 1.0000 | 1.0000 | 1.0000 |  | 1.0000 | 1.0000 | 1.0000 | 1.0000 | 1.0000 | 1.0000 |
| **25** | 0.0000 | 0.2070 | 1.0000 | 0.9013 | 0.9975 | 1.0000 | 1.0000 | 1.0000 | 1.0000 | 1.0000 | 1.0000 | 1.0000 | 1.0000 | 1.0000 | 1.0000 | 1.0000 | 1.0000 | 1.0000 | 1.0000 | 1.0000 | 1.0000 | 1.0000 | 1.0000 | 1.0000 |  | 1.0000 | 1.0000 | 1.0000 | 1.0000 | 1.0000 |
| **26** | 0.0000 | 0.2070 | 1.0000 | 0.9013 | 0.9975 | 1.0000 | 1.0000 | 1.0000 | 1.0000 | 1.0000 | 1.0000 | 1.0000 | 1.0000 | 1.0000 | 1.0000 | 1.0000 | 1.0000 | 1.0000 | 1.0000 | 1.0000 | 1.0000 | 1.0000 | 1.0000 | 1.0000 | 1.0000 |  | 1.0000 | 1.0000 | 1.0000 | 1.0000 |
| **27** | 0.0000 | 0.1686 | 1.0000 | 0.8710 | 0.9958 | 1.0000 | 1.0000 | 1.0000 | 1.0000 | 1.0000 | 1.0000 | 1.0000 | 1.0000 | 1.0000 | 1.0000 | 1.0000 | 1.0000 | 1.0000 | 1.0000 | 1.0000 | 1.0000 | 1.0000 | 1.0000 | 1.0000 | 1.0000 | 1.0000 |  | 1.0000 | 1.0000 | 1.0000 |
| **28** | 0.0000 | 0.0045 | 0.9854 | 0.2535 | 0.7566 | 1.0000 | 0.9909 | 1.0000 | 0.9968 | 0.9982 | 1.0000 | 1.0000 | 1.0000 | 1.0000 | 1.0000 | 1.0000 | 1.0000 | 1.0000 | 1.0000 | 1.0000 | 1.0000 | 1.0000 | 1.0000 | 1.0000 | 1.0000 | 1.0000 | 1.0000 |  | 1.0000 | 1.0000 |
| **29** | 0.0000 | 0.2535 | 1.0000 | 0.9278 | 0.9986 | 1.0000 | 1.0000 | 1.0000 | 1.0000 | 1.0000 | 1.0000 | 1.0000 | 1.0000 | 1.0000 | 1.0000 | 1.0000 | 1.0000 | 1.0000 | 1.0000 | 1.0000 | 1.0000 | 1.0000 | 1.0000 | 1.0000 | 1.0000 | 1.0000 | 1.0000 | 1.0000 |  | 1.0000 |
| **30** | 0.0000 | 0.0519 | 0.9997 | 0.6556 | 0.9670 | 1.0000 | 0.9999 | 1.0000 | 1.0000 | 1.0000 | 1.0000 | 1.0000 | 1.0000 | 1.0000 | 1.0000 | 1.0000 | 1.0000 | 1.0000 | 1.0000 | 1.0000 | 1.0000 | 1.0000 | 1.0000 | 1.0000 | 1.0000 | 1.0000 | 1.0000 | 1.0000 | 1.0000 |  |
