# Supplementary material for: Sleep timing in flies from “adolescence” to adulthood
Source: Fly (Austin). 2024 Dec 30;19(1):2448022. doi: 10.1080/19336934.2024.2448022 (PMC11702927; doi:10.1080/19336934.2024.2448022)
Supplement: FliesAge_Suppl_Table3.docx [file KFLY_A_2448022_SM2378.docx]

| **Day** | **1** | **2** | **3** | **4** | **5** | **6** | **7** | **8** | **9** | **10** | **11** | **12** | **13** | **14** | **15** | **16** | **17** | **18** | **19** | **20** | **21** | **22** | **23** | **24** | **25** | **26** | **27** | **28** | **29** |
| --- | --- | --- | --- | --- | --- | --- | --- | --- | --- | --- | --- | --- | --- | --- | --- | --- | --- | --- | --- | --- | --- | --- | --- | --- | --- | --- | --- | --- | --- |
| **1** |  | 1.0000 | 1.0000 | 1.0000 | 1.0000 | 0.6925 | 0.8345 | 1.0000 | 1.0000 | 1.0000 | 0.3032 | 0.0019 | 0.0007 | 0.0832 | 0.0145 | 0.6249 | 0.2659 | 0.9758 | 0.8871 | 0.3268 | 0.9894 | 0.8162 | 0.6074 | 0.9931 | 0.9125 | 0.7547 | 0.5897 | 0.3268 | 0.6161 |
| **2** | 1.0000 |  | 1.0000 | 1.0000 | 1.0000 | 0.8917 | 0.9577 | 1.0000 | 1.0000 | 1.0000 | 0.5718 | 0.0117 | 0.0053 | 0.2378 | 0.0626 | 0.8517 | 0.5268 | 0.9973 | 0.9758 | 0.5985 | 0.9991 | 0.9505 | 0.8404 | 0.9995 | 0.9833 | 0.9235 | 0.8285 | 0.5985 | 0.8461 |
| **3** | 1.0000 | 1.0000 |  | 1.0000 | 1.0000 | 0.9125 | 0.9678 | 1.0000 | 1.0000 | 1.0000 | 0.6161 | 0.0153 | 0.0071 | 0.2731 | 0.0768 | 0.8776 | 0.5718 | 0.9982 | 0.9822 | 0.6422 | 0.9995 | 0.9620 | 0.8676 | 0.9997 | 0.9879 | 0.9396 | 0.8571 | 0.6422 | 0.8727 |
| **4** | 1.0000 | 1.0000 | 1.0000 |  | 1.0000 | 0.9986 | 0.9998 | 1.0000 | 1.0000 | 1.0000 | 0.9659 | 0.2116 | 0.1346 | 0.8034 | 0.4907 | 0.9973 | 0.9554 | 1.0000 | 0.9999 | 0.9712 | 1.0000 | 0.9997 | 0.9968 | 1.0000 | 1.0000 | 0.9993 | 0.9962 | 0.9712 | 0.9970 |
| **5** | 1.0000 | 1.0000 | 1.0000 | 1.0000 |  | 0.9989 | 0.9999 | 1.0000 | 1.0000 | 1.0000 | 0.9712 | 0.2311 | 0.1492 | 0.8224 | 0.5178 | 0.9979 | 0.9620 | 1.0000 | 1.0000 | 0.9758 | 1.0000 | 0.9998 | 0.9975 | 1.0000 | 1.0000 | 0.9995 | 0.9970 | 0.9758 | 0.9977 |
| **6** | 0.6925 | 0.8917 | 0.9125 | 0.9986 | 0.9989 |  | 1.0000 | 1.0000 | 0.9480 | 0.8727 | 1.0000 | 1.0000 | 1.0000 | 1.0000 | 1.0000 | 1.0000 | 1.0000 | 1.0000 | 1.0000 | 1.0000 | 1.0000 | 1.0000 | 1.0000 | 1.0000 | 1.0000 | 1.0000 | 1.0000 | 1.0000 | 1.0000 |
| **7** | 0.8345 | 0.9577 | 0.9678 | 0.9998 | 0.9999 | 1.0000 |  | 1.0000 | 0.9833 | 0.9480 | 1.0000 | 1.0000 | 0.9998 | 1.0000 | 1.0000 | 1.0000 | 1.0000 | 1.0000 | 1.0000 | 1.0000 | 1.0000 | 1.0000 | 1.0000 | 1.0000 | 1.0000 | 1.0000 | 1.0000 | 1.0000 | 1.0000 |
| **8** | 1.0000 | 1.0000 | 1.0000 | 1.0000 | 1.0000 | 1.0000 | 1.0000 |  | 1.0000 | 1.0000 | 0.9998 | 0.7321 | 0.6161 | 0.9920 | 0.9270 | 1.0000 | 0.9996 | 1.0000 | 1.0000 | 0.9998 | 1.0000 | 1.0000 | 1.0000 | 1.0000 | 1.0000 | 1.0000 | 1.0000 | 0.9998 | 1.0000 |
| **9** | 1.0000 | 1.0000 | 1.0000 | 1.0000 | 1.0000 | 0.9480 | 0.9833 | 1.0000 |  | 1.0000 | 0.7086 | 0.0266 | 0.0130 | 0.3595 | 0.1168 | 0.9235 | 0.6677 | 0.9993 | 0.9914 | 0.7321 | 0.9998 | 0.9798 | 0.9163 | 0.9999 | 0.9945 | 0.9659 | 0.9086 | 0.7321 | 0.9200 |
| **10** | 1.0000 | 1.0000 | 1.0000 | 1.0000 | 1.0000 | 0.8727 | 0.9480 | 1.0000 | 1.0000 |  | 0.5358 | 0.0094 | 0.0042 | 0.2116 | 0.0528 | 0.8285 | 0.4907 | 0.9962 | 0.9695 | 0.5628 | 0.9987 | 0.9396 | 0.8162 | 0.9993 | 0.9786 | 0.9086 | 0.8034 | 0.5628 | 0.8224 |
| **11** | 0.3032 | 0.5718 | 0.6161 | 0.9659 | 0.9712 | 1.0000 | 1.0000 | 0.9998 | 0.7086 | 0.5358 |  | 1.0000 | 1.0000 | 1.0000 | 1.0000 | 1.0000 | 1.0000 | 1.0000 | 1.0000 | 1.0000 | 1.0000 | 1.0000 | 1.0000 | 1.0000 | 1.0000 | 1.0000 | 1.0000 | 1.0000 | 1.0000 |
| **12** | 0.0019 | 0.0117 | 0.0153 | 0.2116 | 0.2311 | 1.0000 | 1.0000 | 0.7321 | 0.0266 | 0.0094 | 1.0000 |  | 1.0000 | 1.0000 | 1.0000 | 1.0000 | 1.0000 | 0.9970 | 0.9998 | 1.0000 | 0.9920 | 1.0000 | 1.0000 | 0.9879 | 0.9997 | 1.0000 | 1.0000 | 1.0000 | 1.0000 |
| **13** | 0.0007 | 0.0053 | 0.0071 | 0.1346 | 0.1492 | 1.0000 | 0.9998 | 0.6161 | 0.0130 | 0.0042 | 1.0000 | 1.0000 |  | 1.0000 | 1.0000 | 1.0000 | 1.0000 | 0.9914 | 0.9993 | 1.0000 | 0.9798 | 0.9998 | 1.0000 | 0.9712 | 0.9988 | 0.9999 | 1.0000 | 1.0000 | 1.0000 |
| **14** | 0.0832 | 0.2378 | 0.2731 | 0.8034 | 0.8224 | 1.0000 | 1.0000 | 0.9920 | 0.3595 | 0.2116 | 1.0000 | 1.0000 | 1.0000 |  | 1.0000 | 1.0000 | 1.0000 | 1.0000 | 1.0000 | 1.0000 | 1.0000 | 1.0000 | 1.0000 | 1.0000 | 1.0000 | 1.0000 | 1.0000 | 1.0000 | 1.0000 |
| **15** | 0.0145 | 0.0626 | 0.0768 | 0.4907 | 0.5178 | 1.0000 | 1.0000 | 0.9270 | 0.1168 | 0.0528 | 1.0000 | 1.0000 | 1.0000 | 1.0000 |  | 1.0000 | 1.0000 | 0.9999 | 1.0000 | 1.0000 | 0.9996 | 1.0000 | 1.0000 | 0.9993 | 1.0000 | 1.0000 | 1.0000 | 1.0000 | 1.0000 |
| **16** | 0.6249 | 0.8517 | 0.8776 | 0.9973 | 0.9979 | 1.0000 | 1.0000 | 1.0000 | 0.9235 | 0.8285 | 1.0000 | 1.0000 | 1.0000 | 1.0000 | 1.0000 |  | 1.0000 | 1.0000 | 1.0000 | 1.0000 | 1.0000 | 1.0000 | 1.0000 | 1.0000 | 1.0000 | 1.0000 | 1.0000 | 1.0000 | 1.0000 |
| **17** | 0.2659 | 0.5268 | 0.5718 | 0.9554 | 0.9620 | 1.0000 | 1.0000 | 0.9996 | 0.6677 | 0.4907 | 1.0000 | 1.0000 | 1.0000 | 1.0000 | 1.0000 | 1.0000 |  | 1.0000 | 1.0000 | 1.0000 | 1.0000 | 1.0000 | 1.0000 | 1.0000 | 1.0000 | 1.0000 | 1.0000 | 1.0000 | 1.0000 |
| **18** | 0.9758 | 0.9973 | 0.9982 | 1.0000 | 1.0000 | 1.0000 | 1.0000 | 1.0000 | 0.9993 | 0.9962 | 1.0000 | 0.9970 | 0.9914 | 1.0000 | 0.9999 | 1.0000 | 1.0000 |  | 1.0000 | 1.0000 | 1.0000 | 1.0000 | 1.0000 | 1.0000 | 1.0000 | 1.0000 | 1.0000 | 1.0000 | 1.0000 |
| **19** | 0.8871 | 0.9758 | 0.9822 | 0.9999 | 1.0000 | 1.0000 | 1.0000 | 1.0000 | 0.9914 | 0.9695 | 1.0000 | 0.9998 | 0.9993 | 1.0000 | 1.0000 | 1.0000 | 1.0000 | 1.0000 |  | 1.0000 | 1.0000 | 1.0000 | 1.0000 | 1.0000 | 1.0000 | 1.0000 | 1.0000 | 1.0000 | 1.0000 |
| **20** | 0.3268 | 0.5985 | 0.6422 | 0.9712 | 0.9758 | 1.0000 | 1.0000 | 0.9998 | 0.7321 | 0.5628 | 1.0000 | 1.0000 | 1.0000 | 1.0000 | 1.0000 | 1.0000 | 1.0000 | 1.0000 | 1.0000 |  | 1.0000 | 1.0000 | 1.0000 | 1.0000 | 1.0000 | 1.0000 | 1.0000 | 1.0000 | 1.0000 |
| **21** | 0.9894 | 0.9991 | 0.9995 | 1.0000 | 1.0000 | 1.0000 | 1.0000 | 1.0000 | 0.9998 | 0.9987 | 1.0000 | 0.9920 | 0.9798 | 1.0000 | 0.9996 | 1.0000 | 1.0000 | 1.0000 | 1.0000 | 1.0000 |  | 1.0000 | 1.0000 | 1.0000 | 1.0000 | 1.0000 | 1.0000 | 1.0000 | 1.0000 |
| **22** | 0.8162 | 0.9505 | 0.9620 | 0.9997 | 0.9998 | 1.0000 | 1.0000 | 1.0000 | 0.9798 | 0.9396 | 1.0000 | 1.0000 | 0.9998 | 1.0000 | 1.0000 | 1.0000 | 1.0000 | 1.0000 | 1.0000 | 1.0000 | 1.0000 |  | 1.0000 | 1.0000 | 1.0000 | 1.0000 | 1.0000 | 1.0000 | 1.0000 |
| **23** | 0.6074 | 0.8404 | 0.8676 | 0.9968 | 0.9975 | 1.0000 | 1.0000 | 1.0000 | 0.9163 | 0.8162 | 1.0000 | 1.0000 | 1.0000 | 1.0000 | 1.0000 | 1.0000 | 1.0000 | 1.0000 | 1.0000 | 1.0000 | 1.0000 | 1.0000 |  | 1.0000 | 1.0000 | 1.0000 | 1.0000 | 1.0000 | 1.0000 |
| **24** | 0.9931 | 0.9995 | 0.9997 | 1.0000 | 1.0000 | 1.0000 | 1.0000 | 1.0000 | 0.9999 | 0.9993 | 1.0000 | 0.9879 | 0.9712 | 1.0000 | 0.9993 | 1.0000 | 1.0000 | 1.0000 | 1.0000 | 1.0000 | 1.0000 | 1.0000 | 1.0000 |  | 1.0000 | 1.0000 | 1.0000 | 1.0000 | 1.0000 |
| **25** | 0.9125 | 0.9833 | 0.9879 | 1.0000 | 1.0000 | 1.0000 | 1.0000 | 1.0000 | 0.9945 | 0.9786 | 1.0000 | 0.9997 | 0.9988 | 1.0000 | 1.0000 | 1.0000 | 1.0000 | 1.0000 | 1.0000 | 1.0000 | 1.0000 | 1.0000 | 1.0000 | 1.0000 |  | 1.0000 | 1.0000 | 1.0000 | 1.0000 |
| **26** | 0.7547 | 0.9235 | 0.9396 | 0.9993 | 0.9995 | 1.0000 | 1.0000 | 1.0000 | 0.9659 | 0.9086 | 1.0000 | 1.0000 | 0.9999 | 1.0000 | 1.0000 | 1.0000 | 1.0000 | 1.0000 | 1.0000 | 1.0000 | 1.0000 | 1.0000 | 1.0000 | 1.0000 | 1.0000 |  | 1.0000 | 1.0000 | 1.0000 |
| **27** | 0.5897 | 0.8285 | 0.8571 | 0.9962 | 0.9970 | 1.0000 | 1.0000 | 1.0000 | 0.9086 | 0.8034 | 1.0000 | 1.0000 | 1.0000 | 1.0000 | 1.0000 | 1.0000 | 1.0000 | 1.0000 | 1.0000 | 1.0000 | 1.0000 | 1.0000 | 1.0000 | 1.0000 | 1.0000 | 1.0000 |  | 1.0000 | 1.0000 |
| **28** | 0.3268 | 0.5985 | 0.6422 | 0.9712 | 0.9758 | 1.0000 | 1.0000 | 0.9998 | 0.7321 | 0.5628 | 1.0000 | 1.0000 | 1.0000 | 1.0000 | 1.0000 | 1.0000 | 1.0000 | 1.0000 | 1.0000 | 1.0000 | 1.0000 | 1.0000 | 1.0000 | 1.0000 | 1.0000 | 1.0000 | 1.0000 |  | 1.0000 |
| **29** | 0.6161 | 0.8461 | 0.8727 | 0.9970 | 0.9977 | 1.0000 | 1.0000 | 1.0000 | 0.9200 | 0.8224 | 1.0000 | 1.0000 | 1.0000 | 1.0000 | 1.0000 | 1.0000 | 1.0000 | 1.0000 | 1.0000 | 1.0000 | 1.0000 | 1.0000 | 1.0000 | 1.0000 | 1.0000 | 1.0000 | 1.0000 | 1.0000 |  |

**Supplementary Table 3A***. post hoc* Scheffé test (p<0.05 marked in red) for number of sleep bouts over time (days) during the *lights off* interval.

| **Day** | **1** | **2** | **3** | **4** | **5** | **6** | **7** | **8** | **9** | **10** | **11** | **12** | **13** | **14** | **15** | **16** | **17** | **18** | **19** | **20** | **21** | **22** | **23** | **24** | **25** | **26** | **27** | **28** | **29** |
| --- | --- | --- | --- | --- | --- | --- | --- | --- | --- | --- | --- | --- | --- | --- | --- | --- | --- | --- | --- | --- | --- | --- | --- | --- | --- | --- | --- | --- | --- |
| **1** |  | 1.0000 | 1.0000 | 1.0000 | 1.0000 | 1.0000 | 1.0000 | 1.0000 | 1.0000 | 1.0000 | 0.9947 | 0.5209 | 0.0000 | 0.5075 | 0.1404 | 0.9620 | 0.2022 | 0.9999 | 0.6394 | 0.9798 | 1.0000 | 0.9995 | 0.9894 | 0.9979 | 0.8987 | 0.9925 | 0.9836 | 0.9898 | 0.9997 |
| **2** | 1.0000 |  | 1.0000 | 1.0000 | 1.0000 | 1.0000 | 1.0000 | 1.0000 | 1.0000 | 1.0000 | 0.9972 | 0.5929 | 0.0000 | 0.5797 | 0.1829 | 0.9759 | 0.2552 | 1.0000 | 0.7063 | 0.9879 | 1.0000 | 0.9998 | 0.9940 | 0.9990 | 0.9291 | 0.9959 | 0.9904 | 0.9943 | 0.9999 |
| **3** | 1.0000 | 1.0000 |  | 1.0000 | 1.0000 | 1.0000 | 1.0000 | 1.0000 | 1.0000 | 1.0000 | 1.0000 | 0.9568 | 0.0027 | 0.9534 | 0.6885 | 0.9999 | 0.7746 | 1.0000 | 0.9798 | 1.0000 | 1.0000 | 1.0000 | 1.0000 | 1.0000 | 0.9989 | 1.0000 | 1.0000 | 1.0000 | 1.0000 |
| **4** | 1.0000 | 1.0000 | 1.0000 |  | 1.0000 | 1.0000 | 1.0000 | 1.0000 | 1.0000 | 1.0000 | 0.9999 | 0.8791 | 0.0006 | 0.8718 | 0.4924 | 0.9987 | 0.5937 | 1.0000 | 0.9324 | 0.9996 | 1.0000 | 1.0000 | 0.9998 | 1.0000 | 0.9934 | 0.9999 | 0.9997 | 0.9999 | 1.0000 |
| **5** | 1.0000 | 1.0000 | 1.0000 | 1.0000 |  | 1.0000 | 1.0000 | 1.0000 | 1.0000 | 1.0000 | 1.0000 | 0.8983 | 0.0008 | 0.8918 | 0.5304 | 0.9991 | 0.6306 | 1.0000 | 0.9449 | 0.9997 | 1.0000 | 1.0000 | 0.9999 | 1.0000 | 0.9952 | 0.9999 | 0.9998 | 0.9999 | 1.0000 |
| **6** | 1.0000 | 1.0000 | 1.0000 | 1.0000 | 1.0000 |  | 1.0000 | 1.0000 | 1.0000 | 1.0000 | 1.0000 | 1.0000 | 0.2201 | 1.0000 | 0.9978 | 1.0000 | 0.9992 | 1.0000 | 1.0000 | 1.0000 | 1.0000 | 1.0000 | 1.0000 | 1.0000 | 1.0000 | 1.0000 | 1.0000 | 1.0000 | 1.0000 |
| **7** | 1.0000 | 1.0000 | 1.0000 | 1.0000 | 1.0000 | 1.0000 |  | 1.0000 | 1.0000 | 1.0000 | 1.0000 | 0.9999 | 0.1057 | 0.9999 | 0.9885 | 1.0000 | 0.9949 | 1.0000 | 1.0000 | 1.0000 | 1.0000 | 1.0000 | 1.0000 | 1.0000 | 1.0000 | 1.0000 | 1.0000 | 1.0000 | 1.0000 |
| **8** | 1.0000 | 1.0000 | 1.0000 | 1.0000 | 1.0000 | 1.0000 | 1.0000 |  | 1.0000 | 1.0000 | 1.0000 | 0.9953 | 0.0192 | 0.9947 | 0.9043 | 1.0000 | 0.9431 | 1.0000 | 0.9984 | 1.0000 | 1.0000 | 1.0000 | 1.0000 | 1.0000 | 1.0000 | 1.0000 | 1.0000 | 1.0000 | 1.0000 |
| **9** | 1.0000 | 1.0000 | 1.0000 | 1.0000 | 1.0000 | 1.0000 | 1.0000 | 1.0000 |  | 1.0000 | 1.0000 | 0.9530 | 0.0025 | 0.9493 | 0.6750 | 0.9998 | 0.7630 | 1.0000 | 0.9777 | 1.0000 | 1.0000 | 1.0000 | 1.0000 | 1.0000 | 0.9988 | 1.0000 | 1.0000 | 1.0000 | 1.0000 |
| **10** | 1.0000 | 1.0000 | 1.0000 | 1.0000 | 1.0000 | 1.0000 | 1.0000 | 1.0000 | 1.0000 |  | 0.9997 | 0.7801 | 0.0002 | 0.7697 | 0.3465 | 0.9949 | 0.4432 | 1.0000 | 0.8618 | 0.9979 | 1.0000 | 1.0000 | 0.9991 | 0.9999 | 0.9796 | 0.9995 | 0.9984 | 0.9992 | 1.0000 |
| **11** | 0.9947 | 0.9972 | 1.0000 | 0.9999 | 1.0000 | 1.0000 | 1.0000 | 1.0000 | 1.0000 | 0.9997 |  | 1.0000 | 0.7256 | 1.0000 | 1.0000 | 1.0000 | 1.0000 | 1.0000 | 1.0000 | 1.0000 | 1.0000 | 1.0000 | 1.0000 | 1.0000 | 1.0000 | 1.0000 | 1.0000 | 1.0000 | 1.0000 |
| **12** | 0.5209 | 0.5929 | 0.9568 | 0.8791 | 0.8983 | 1.0000 | 0.9999 | 0.9953 | 0.9530 | 0.7801 | 1.0000 |  | 0.9993 | 1.0000 | 1.0000 | 1.0000 | 1.0000 | 1.0000 | 1.0000 | 1.0000 | 0.9999 | 1.0000 | 1.0000 | 1.0000 | 1.0000 | 1.0000 | 1.0000 | 1.0000 | 1.0000 |
| **13** | 0.0000 | 0.0000 | 0.0027 | 0.0006 | 0.0008 | 0.2201 | 0.1057 | 0.0192 | 0.0025 | 0.0002 | 0.7256 | 0.9993 |  | 0.9994 | 1.0000 | 0.9074 | 1.0000 | 0.3126 | 0.9977 | 0.8558 | 0.1309 | 0.4971 | 0.7952 | 0.6282 | 0.9661 | 0.7604 | 0.8370 | 0.7915 | 0.4353 |
| **14** | 0.5075 | 0.5797 | 0.9534 | 0.8718 | 0.8918 | 1.0000 | 0.9999 | 0.9947 | 0.9493 | 0.7697 | 1.0000 | 1.0000 | 0.9994 |  | 1.0000 | 1.0000 | 1.0000 | 1.0000 | 1.0000 | 1.0000 | 0.9999 | 1.0000 | 1.0000 | 1.0000 | 1.0000 | 1.0000 | 1.0000 | 1.0000 | 1.0000 |
| **15** | 0.1404 | 0.1829 | 0.6885 | 0.4924 | 0.5304 | 0.9978 | 0.9885 | 0.9043 | 0.6750 | 0.3465 | 1.0000 | 1.0000 | 1.0000 | 1.0000 |  | 1.0000 | 1.0000 | 0.9993 | 1.0000 | 1.0000 | 0.9924 | 0.9999 | 1.0000 | 1.0000 | 1.0000 | 1.0000 | 1.0000 | 1.0000 | 0.9998 |
| **16** | 0.9620 | 0.9759 | 0.9999 | 0.9987 | 0.9991 | 1.0000 | 1.0000 | 1.0000 | 0.9998 | 0.9949 | 1.0000 | 1.0000 | 0.9074 | 1.0000 | 1.0000 |  | 1.0000 | 1.0000 | 1.0000 | 1.0000 | 1.0000 | 1.0000 | 1.0000 | 1.0000 | 1.0000 | 1.0000 | 1.0000 | 1.0000 | 1.0000 |
| **17** | 0.2022 | 0.2552 | 0.7746 | 0.5937 | 0.6306 | 0.9992 | 0.9949 | 0.9431 | 0.7630 | 0.4432 | 1.0000 | 1.0000 | 1.0000 | 1.0000 | 1.0000 | 1.0000 |  | 0.9998 | 1.0000 | 1.0000 | 0.9968 | 1.0000 | 1.0000 | 1.0000 | 1.0000 | 1.0000 | 1.0000 | 1.0000 | 1.0000 |
| **18** | 0.9999 | 1.0000 | 1.0000 | 1.0000 | 1.0000 | 1.0000 | 1.0000 | 1.0000 | 1.0000 | 1.0000 | 1.0000 | 1.0000 | 0.3126 | 1.0000 | 0.9993 | 1.0000 | 0.9998 |  | 1.0000 | 1.0000 | 1.0000 | 1.0000 | 1.0000 | 1.0000 | 1.0000 | 1.0000 | 1.0000 | 1.0000 | 1.0000 |
| **19** | 0.6394 | 0.7063 | 0.9798 | 0.9324 | 0.9449 | 1.0000 | 1.0000 | 0.9984 | 0.9777 | 0.8618 | 1.0000 | 1.0000 | 0.9977 | 1.0000 | 1.0000 | 1.0000 | 1.0000 | 1.0000 |  | 1.0000 | 1.0000 | 1.0000 | 1.0000 | 1.0000 | 1.0000 | 1.0000 | 1.0000 | 1.0000 | 1.0000 |
| **20** | 0.9798 | 0.9879 | 1.0000 | 0.9996 | 0.9997 | 1.0000 | 1.0000 | 1.0000 | 1.0000 | 0.9979 | 1.0000 | 1.0000 | 0.8558 | 1.0000 | 1.0000 | 1.0000 | 1.0000 | 1.0000 | 1.0000 |  | 1.0000 | 1.0000 | 1.0000 | 1.0000 | 1.0000 | 1.0000 | 1.0000 | 1.0000 | 1.0000 |
| **21** | 1.0000 | 1.0000 | 1.0000 | 1.0000 | 1.0000 | 1.0000 | 1.0000 | 1.0000 | 1.0000 | 1.0000 | 1.0000 | 0.9999 | 0.1309 | 0.9999 | 0.9924 | 1.0000 | 0.9968 | 1.0000 | 1.0000 | 1.0000 |  | 1.0000 | 1.0000 | 1.0000 | 1.0000 | 1.0000 | 1.0000 | 1.0000 | 1.0000 |
| **22** | 0.9995 | 0.9998 | 1.0000 | 1.0000 | 1.0000 | 1.0000 | 1.0000 | 1.0000 | 1.0000 | 1.0000 | 1.0000 | 1.0000 | 0.4971 | 1.0000 | 0.9999 | 1.0000 | 1.0000 | 1.0000 | 1.0000 | 1.0000 | 1.0000 |  | 1.0000 | 1.0000 | 1.0000 | 1.0000 | 1.0000 | 1.0000 | 1.0000 |
| **23** | 0.9894 | 0.9940 | 1.0000 | 0.9998 | 0.9999 | 1.0000 | 1.0000 | 1.0000 | 1.0000 | 0.9991 | 1.0000 | 1.0000 | 0.7952 | 1.0000 | 1.0000 | 1.0000 | 1.0000 | 1.0000 | 1.0000 | 1.0000 | 1.0000 | 1.0000 |  | 1.0000 | 1.0000 | 1.0000 | 1.0000 | 1.0000 | 1.0000 |
| **24** | 0.9979 | 0.9990 | 1.0000 | 1.0000 | 1.0000 | 1.0000 | 1.0000 | 1.0000 | 1.0000 | 0.9999 | 1.0000 | 1.0000 | 0.6282 | 1.0000 | 1.0000 | 1.0000 | 1.0000 | 1.0000 | 1.0000 | 1.0000 | 1.0000 | 1.0000 | 1.0000 |  | 1.0000 | 1.0000 | 1.0000 | 1.0000 | 1.0000 |
| **25** | 0.8987 | 0.9291 | 0.9989 | 0.9934 | 0.9952 | 1.0000 | 1.0000 | 1.0000 | 0.9988 | 0.9796 | 1.0000 | 1.0000 | 0.9661 | 1.0000 | 1.0000 | 1.0000 | 1.0000 | 1.0000 | 1.0000 | 1.0000 | 1.0000 | 1.0000 | 1.0000 | 1.0000 |  | 1.0000 | 1.0000 | 1.0000 | 1.0000 |
| **26** | 0.9925 | 0.9959 | 1.0000 | 0.9999 | 0.9999 | 1.0000 | 1.0000 | 1.0000 | 1.0000 | 0.9995 | 1.0000 | 1.0000 | 0.7604 | 1.0000 | 1.0000 | 1.0000 | 1.0000 | 1.0000 | 1.0000 | 1.0000 | 1.0000 | 1.0000 | 1.0000 | 1.0000 | 1.0000 |  | 1.0000 | 1.0000 | 1.0000 |
| **27** | 0.9836 | 0.9904 | 1.0000 | 0.9997 | 0.9998 | 1.0000 | 1.0000 | 1.0000 | 1.0000 | 0.9984 | 1.0000 | 1.0000 | 0.8370 | 1.0000 | 1.0000 | 1.0000 | 1.0000 | 1.0000 | 1.0000 | 1.0000 | 1.0000 | 1.0000 | 1.0000 | 1.0000 | 1.0000 | 1.0000 |  | 1.0000 | 1.0000 |
| **28** | 0.9898 | 0.9943 | 1.0000 | 0.9999 | 0.9999 | 1.0000 | 1.0000 | 1.0000 | 1.0000 | 0.9992 | 1.0000 | 1.0000 | 0.7915 | 1.0000 | 1.0000 | 1.0000 | 1.0000 | 1.0000 | 1.0000 | 1.0000 | 1.0000 | 1.0000 | 1.0000 | 1.0000 | 1.0000 | 1.0000 | 1.0000 |  | 1.0000 |
| **29** | 0.9997 | 0.9999 | 1.0000 | 1.0000 | 1.0000 | 1.0000 | 1.0000 | 1.0000 | 1.0000 | 1.0000 | 1.0000 | 1.0000 | 0.4353 | 1.0000 | 0.9998 | 1.0000 | 1.0000 | 1.0000 | 1.0000 | 1.0000 | 1.0000 | 1.0000 | 1.0000 | 1.0000 | 1.0000 | 1.0000 | 1.0000 | 1.0000 |  |

**Supplementary Table 3B***. post hoc* Scheffé test (p<0.05 marked in red) for average sleep bout duration over time (days) during the *lights off* interval.
