# Supplementary material for: Sleep timing in flies from “adolescence” to adulthood
Source: Fly (Austin). 2024 Dec 30;19(1):2448022. doi: 10.1080/19336934.2024.2448022 (PMC11702927; doi:10.1080/19336934.2024.2448022)
Supplement: FliesAge_Suppl_Figures1_and_2.docx [file KFLY_A_2448022_SM2376.docx]

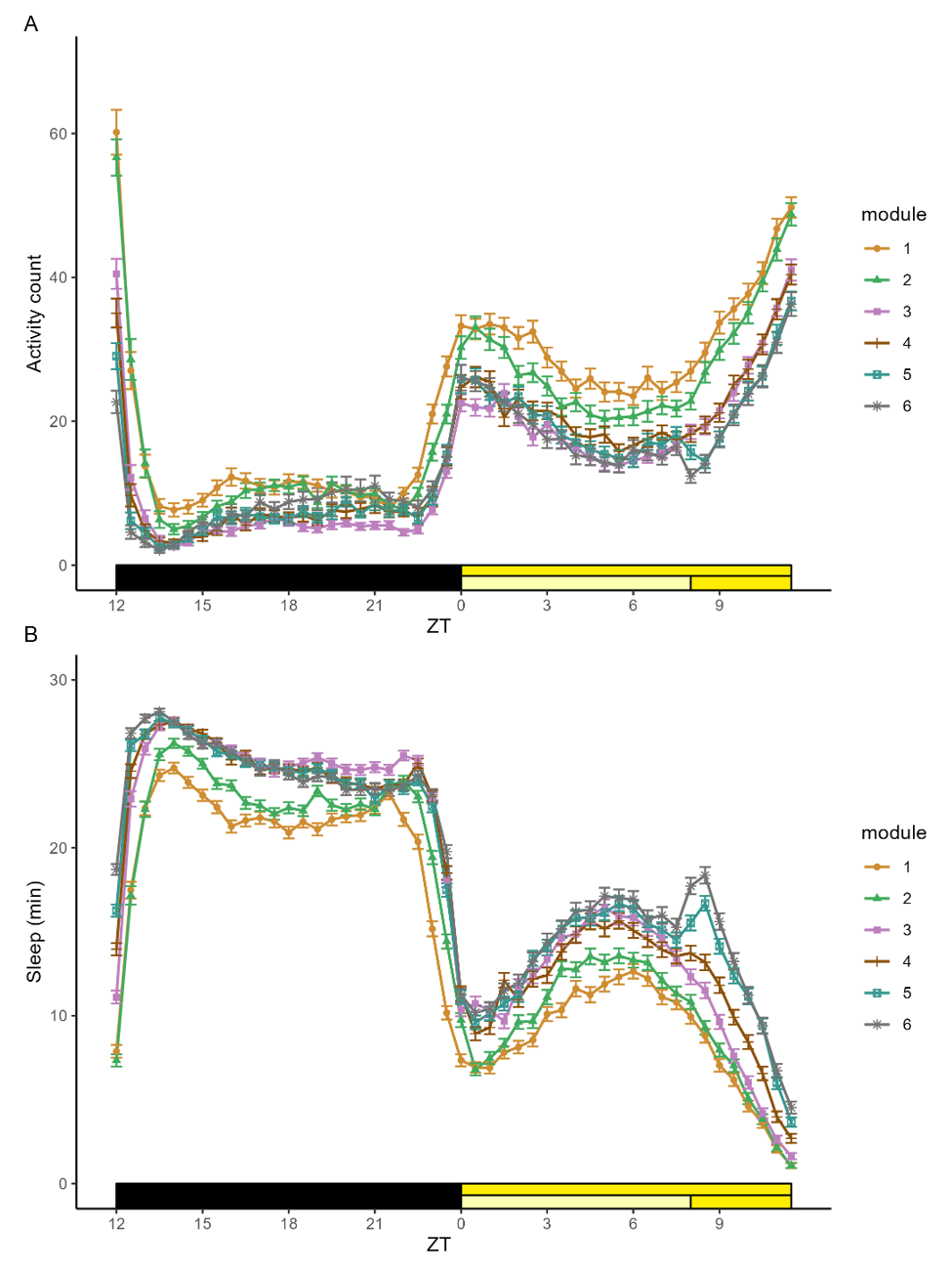


**Supplementary Figure 1. Activity (A) and sleep (B) profiles, by module.** Activity counts (A, mean ± SEM) and minutes of sleep per 30 minutes (B, mean ± SEM) over 24 hours, by module, including both working and free days in n=113 flies. The light:dark schedule for working (bottom bar) and free days (top bar) is indicated on top of the x axis (black: darkness; light yellow: 230 lux; bright yellow: 900 lux).


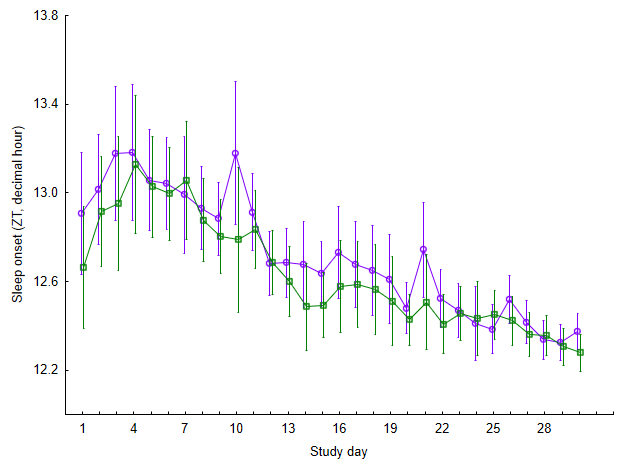


**Supplementary Figure 2A. Sleep onset over time,** by day, in the two random subsamples (green and purple). *Repeated measures ANOVA, by group: time: F=21, p=0.0001; group: F=1, p=0.305; time*group (interaction): F=0.8, p=0.799.*


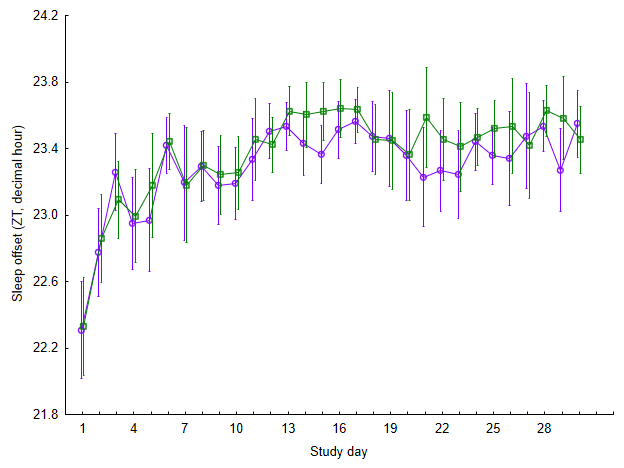


**Supplementary Figure 2B. Sleep offset over time**, by day, in the two random subsamples (green and purple). *Repeated measures ANOVA, by group: time: F=13, p=0.0001; group: F=0.9, p=0.352; time*group (interaction): F=0.6, p=0.924.*
